# Novel Hydroxytyrosol-Donepezil Hybrids as Potential Antioxidant and Neuroprotective Agents

**DOI:** 10.3389/fchem.2021.741444

**Published:** 2021-10-19

**Authors:** Paola Costanzo, Manuela Oliverio, Jessica Maiuolo, Sonia Bonacci, Giuseppina De Luca, Mariorosario Masullo, Rosaria Arcone, Antonio Procopio

**Affiliations:** ^1^ Dipartimento di Chimica e Tecnologie Chimiche, Università della Calabria, Rende, Italy; ^2^ Dipartimento di Scienze della Salute, Università Magna Græcia di Catanzaro, Catanzaro, Italy; ^3^ Dipartimento di Scienze Motorie e del Benessere, Università degli Studi di Napoli “Parthenope”, Napoli, Italy; ^4^ CEINGE Biotecnologie Avanzate S.C.a R.L., Napoli, Italy

**Keywords:** antioxidant, bio-phenols, neurodegeneration, bio-metal chelator, reactive oxygen species

## Abstract

It is well-accepted that the endogenous antioxidant protection system progressively decays in elderly people, and that the oxidative stress contributes to different neurodegenerative disorders such as Alzheimer’s Diseases (AD). The lower incidence of AD in countries which feature the Mediterranean Diet was associated to the high consumption of extra virgin olive oil and its polyphenolic fraction, in particular hydroxytyrosol. The protective role of these bio-phenols against oxidative stress, suggested that we combine their antioxidant/free radical scavenging activity with donepezil, an active ingredient which has just been approved for the treatment of AD. Different synthetic strategies were tested to conjugate the two different synthons in good yields. Additionally, a nitro-hydroxytyrosol derivative was synthesized to extend the application to other neurodegeneration inflammatory models. Then, their bioactivity was measured in different chemical and biological tests on a human neuroblastoma cell line (SHSY-5Y). Remarkable results on cell viability and the regulation of the redox state of cells were obtained. All hybrids showed negligible cell death under 1 μM and are stable and non toxic. Reactive oxygen species (ROS) measurements showed that the nitro-hybrid was the more effective one at reducing the ROS amount to physiological values. Then, in light of the bio-metal hypothesis of diverse neurodegenerative disorders, we tested these new compounds on the chelation properties of redox-active metals. The nitro-hybrid was able to chelate all of the tested metal cations, suggesting that we propose it as potential lead compound for a new class of neuroprotective antioxidant agents.

## 1 Introduction

**FIGURE 1:**
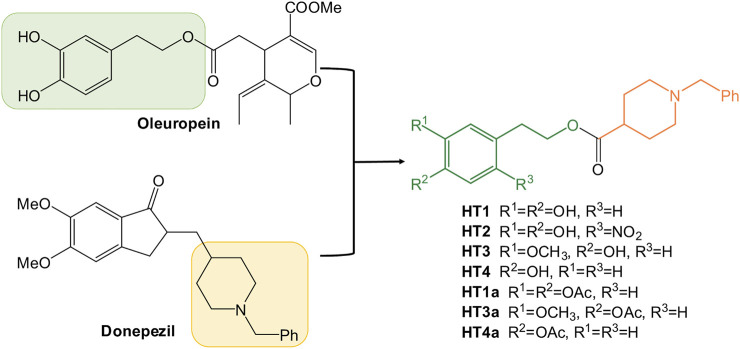
Development of new hydroxytyrosol-donepezil hybrids.

With longer life expectancies and aging thanks to the modern medical advances as well as social and environmental conditions, neurological disorders are increasing. Neurodegenerative diseases, as other age-related conditions such as diabetes, cardiovascular diseases, sarcopenia, and cancer are also associated with the so-called “oxidative stress theory of aging” (Liguori et al., 2018). In physiological conditions, the production of free radicals like the reactive oxygen species (ROS) is counterbalanced by the antioxidant systems, but in pathological conditions, or elderly peoples, the antioxidant defenses are not still to balance the ROS formation anymore. For these reasons many efforts have been made to develop new drugs with antioxidant and scavenging activities (Maiuolo et al., 2017; Neha et al., 2019). In particular, the excessive production of ROS contributing to oxidative stress leads to neuronal cell death and altered brain function (Singh et al., 2019). The greater sensitivity of the brain to oxidative stress could be explained by its high oxygen demand and consumption, as well as the presence of redox-active metals, such as iron and copper, which is increased in the brains of Alzheimer’s disease (AD) and Parkinson’s disease (PD) patients (Barnham and Bush, 2008). During the last decades many reviews and papers have demonstrated the role of oxidative stress in the pathogenesis of these neurodegenerative diseases, and the administration of both natural and synthetic antioxidant molecules was studied in diverse clinical trials (Blesa et al., 2015; Kim et al., 2015; Ganguly et al., 2021). The beneficial effects of the Mediterranean diet against age-related cognitive decline have been highlighted (Gardener and Caunca, 2018; Balakrishnan et al., 2021), and they were associated to the high consumption of olive oil (Kalatsbari, 2013). It was recently revealed that the poly(phenolic) components of extra virgin olive oil could be neuroprotective and ameliorative against these diseases, by working on several clinical biomarkers and molecular targets (Spagnuolo et al., 2016; Dinda et al., 2019). The beneficial health effects of the phenolic fraction in virgin and/or extra virgin olive oil are due to the secoiridoids oleuropein and ligstroside, together with its derivatives: aglycones (Leri et al., 2018), oleocanthal (Batarseh et al., 2018), oleacein (Cuyàs et al., 2019), tyrosol and hydroxytyrosol (St-Laurent-Thibault et al., 2011; Bulotta et al., 2013; Crespo et al., 2017). Different mechanisms for the neuroprotective action of these natural compounds were hypothesized. The main mechanisms studied were free radical scavenging/antioxidant actions, anti-inflammatory effects, and antiapoptotic properties (Kalatsbari, 2013). Furthermore, the recent literature showed an important effect of different nitro-hydroxytyrosol derivatives on PD inflammatory models, demonstrating that the antioxidant properties of the ortho-diphenol moiety could be extended with the right modifications (Gallardo et al., 2014). And considering the multifactorial origins of these disorders, the study of nature inspired multitarget direct ligands with antioxidant and biometal chelating activity remains of interest (Costa et al., 2020; Cui et al., 2020). Donepezil, the second drug approved by the FDA for the treatment of AD, is the most commonly used pharmacophore for the design of novel derivatives, with a potential multifunctional activity (Unzeta et al., 2016; Li et al., 2018). In the last few years, the *N*-benzylpiperidine synthon was combined with natural compounds such as ferulic acid or curcumine (Xu et al., 2016; Dia et al., 2017) in order to obtain a new series of donepezil hybrids with potent cholinesterase inhibitory activities, radical scavenging activities and metal-chelating properties. Then, based on our experiences on the green manipulation of oleuropein and its derivatives (Oliverio et al., 2016; Costanzo et al., 2018; Cariati et al., 2019; Oliverio et al., 2021) and the sustainable synthesis of bioactive compounds (Costanzo et al., 2016; Caliandro et al., 2018), we decided to investigate the neuroprotective features of the olive oil polyphenols as multitarget drug candidates for the treatment of Alzheimer’s disease. Herein, we reported the design, synthesis, characterization and biological tests of new donepezil hybrids with hydroxytyrosol (Figure 1). Furthermore, in order to extend the cognitive effect of these donepezil derivatives, a nitro-hydroxytyrosol (NO-HT) synthon was also combined into the donepezil structure.

## 2 Materials and Methods

### 2.1 Chemistry

#### 2.1.1 General Information

All chemicals were obtained from Sigma Aldrich and Alfa Aesar, and used as received. Hydroxytyrosol was obtained as reported in literature (Gambacorta et al., 2007). MW-assisted reactions, performed in the high energy density laboratory CEM Discover Microwave were run on dynamic mode, applying a specified amount of power to reach the desired temperature and pressure, working with borosilicate glass vessels equipped with silicon cap with septum. Reactions were monitored by TLC using silica plates 60-F264 on alumina, commercially available from Merk. Liquid flash chromatography was performed on a Supelco VERSA FLASH HTFP station using silica cartridges commercially available from Supelco. ^1^H-NMR and ^13^C-NMR spectra were recorded on a Bruker WM 300 instrument on samples dissolved in CDCl_3_(compounds **HT1a, HT3, HT4, HT3a, HT4a**) or in d_6_-DMSO (compounds **HT1** and **HT2**). Chemical shifts are given in parts per million (ppm) from tetramethylsilane as the internal standard (0.0 ppm). HRMS measurements were realized on a Thermo Scientific QExactive (Thermo Fisher, Milan, Italy) mass spectrometer working in positive mode at 35,000 resolving power, operating in SIM mode by flow injection (flow rate 15 μl/min for each stock solution). Stock solutions at a concentration of 1 mg/ml were prepared in UHPLC-MS grade MeOH for each analyte separately. Prior to analysis, each solution was diluted 1:1,000 (v/v) in a vial to obtain a concentration of 1 mg/L. Detection of the targeted compounds was based on theoretical exact mass. Data were evaluated by Xcalibur 2.2.SP1 (Thermo Fisher Scientific, Bremen, Germany). The mass accuracy, directly calculated from Xcalibur, is defined by the formula Δ(ppm) = [(theoretical mass − measured mass)/theoretical mass] × 1.000.000. The ORAC assay employed a microplate fluorimeter (Victor 2 multilabel counter, PerkinElmer Life Sciences, Waltham, MA, United States; excitation at 495 nm; emission at 535 nm; 0.5 s per reading from the bottom of the plate; 1 reading per min) and 24-well plates. Absorption measurements for the CUPRAC assay and the metal-chelating properties characterization were performed on a Perkin-Elmer Lambda 35 spectrophotometer.

#### 2.1.2 General Protocol for the Synthesis of *N*-Benzylpiperidine-4-Carboxylic Acid

The method for the ester hydrolysis was adapted from the procedure developed by Theodorou et al. (2007). Ethyl *N*-benzylpiperidine-4-carboxylate (1.08 g, 4.37 mmol) was dissolved in 20 ml of dichloromethane:methanol (9:1). Then, 3.0 ml of NaOH in methanol (2 N) were added, and the mixture is reacted for 24 h at room temperature and monitored by TLC. After 24 h, 1.0 ml of the basic solution was added, and the mixture was stirred for another 24 h. At the end of the reaction, the solvent was removed under reduced pressure, the residue was diluted with water and extracted three times with diethyl ether. The water phases were collected, cooled at 0°C, and acidified with HCl (6 N) till pH = 2 ÷ 3. Finally, the water phase was extracted three times with ethyl acetate. The organic phases were collected, dried with Na_2_SO_4_, filtered, and evaporated under reduced pressure. The product was used without further purification procedures.

#### 2.1.3 General Protocol for the Synthesis of the Donepezil Hybrids

The best reaction conditions developed for the synthesis of donepezil hybrids are described below. To a solution of *N*-benzylpiperidine-4-carboxylic acid (50 mg, 0.23 mmol) THF (5 ml), the desired polyphenolic alcohol (10 equivalents) and trimethylsilyl chloride (CTMS, 2.5 equivalents) were added in a 2-necked round bottom flask equipped with a magnetic stirrer and a condenser. The mixture was heated at 40°C for 6 days. Subsequently, the solvent was removed under reduced pressure, the crude was diluted in dichloromethane and was extracted three times with NaHCO_3_ (saturated solution). The organic phases were collected and dried with Na_2_SO_4_, filtered, and evaporated under reduced pressure. The product is separated from the crude by flash chromatography (CH_2_Cl_2_/MeOH 9.5:0.5 v/v). All compounds (**HT1**, **HT3**, and **HT4**) were identified by HRMS and characterized by ^1^H-NMR and ^13^C-NMR (See Supplementary Material).

The microwave-assisted synthesis of the acetylated compounds (**HT1a**, **HT3a**, and **HT4a**) were done as reported in literature (Oliverio et al., 2016). All compounds were identified by HRMS and characterized by ^1^H-NMR and ^13^C-NMR (See Supplementary Material).

The synthesis of the nitro-derivative was obtained with the procedure reported in literature (Trujillo et al., 2014). Briefly, **HT1** (55 mg, 0.155 mmol) was dissolved in AcOH/AcONa buffer (pH = 3.75, 31 ml). NaNO_2_ (21.4 mg, 0.31 mmol) was added, and the mixture was stirred at room temperature in the dark for 30 min. Then, the aqueous solution was extracted three times with ethyl acetate. The organic phases were collected and dried with Na_2_SO_4_, filtered, and evaporated under reduced pressure. The product is separated from the crude by flash chromatography (CH_2_Cl_2_/MeOH/TEA 9.5:0.4:0.1 v/v).

### 2.2 *In Vitro* Antioxidant Activities

#### 2.2.1 Measurement of Oxygen Radical Absorbance Capacity

The Orac assay was performed as reported in literature (Nardi et al., 2017) with slight modifications on the solvent solution. Briefly, 7% randomly methylated β-cyclodextrin (RMCD) solution (acetone/water/DMSO, 49.5:49.5:1 v/v) was used as the solvent for the Trolox standards and as a blank. The unprotected donepezil hybrids (10 μM) in this 7% RMCD solution were used as samples. To each well, 80 μl of sample, blank or Trolox (1.25, 6.25, 12.5, 25, 50 and 100 μM in 7% RMCD solution) were added to 800 μl of disodium fluorescein solution (FL, 14 μM in PBS, pre-incubated at 37°C for 15 min). Each solution was analysed in duplicate in a “forward-then-reverse” order as described in the literature (Ou et al., 2001). Reactions were initiated by the addition of 300 μl of AAPH (31.7 mM in PBS). The measurement temperature was set at 37°C. A regression equation was built by comparing the net area under the FL decay curve and the Trolox concentration according to the following equation:

Y(µM Trolox)=a+bX(AUC−n)

and the final ORAC_FL_ values were expressed as Trolox equivalents (μmol g^−1^). The area under the curve was calculated with the following equation:

$$\text{AUC}=1+\overset{i=45}{\underset{i=1}{\sum }}f_{1}/f_{0}$$

#### 2.2.2 Cupric Ion Reducing Antioxidant Capacity Method

The CUPRAC test was performed as reported by Ozyürek et al. (2008) on the new compounds in the unacetylated form.

##### Preparation of Solutions

CuCl_2_ solution 10 mM, was prepared by dissolving 0.336.1 g CuCl_2_ in water, and diluting to 250 ml. Neocuproine (Nc) solution 7.5 mM, was prepared by dissolving 15 mg of neocuproine in 10 ml of absolute EtOH. Ammonium acetate 1.0 M was prepared by dissolving 19.27 g of NH_4_Ac in 250 ml of water. The cyclodextrin (2% M-β-CD) stock solution was prepared in water-acetone (9:1 v/v). The standard solution of Trolox 1 mM was prepared in 2% M-β-CD. All working solutions of antioxidant 1 mM were freshly prepared in 2% M-β-CD.

1 ml of Cu(II) + 1 ml of Nc + 1 ml of NH_4_Ac buffer + *x* mL of antioxidant solution + (1.1 − *x*) 2% M-β-CD in 1:9 water–acetone mixture (v/v); total volume = 4.1 ml, measure *A*_450_ against a reagent blank after 40 min of reagent addition at room temperature.

The Trolox equivalent antioxidant capacity (TEAC) is defined as the millimolar concentration of a Trolox solution having the antioxidant capacity equivalent to a 1.0 mM solution of the substance under investigation. The TEAC_CUPRAC_ values were simply calculated by dividing the molar absorptivity ε of the species under investigation by that of Trolox under corresponding conditions. The concentration linearity ranges for all the antioxidants tested are reported in the Supplementary Material (See Supplementary Table S1).

### 2.3 Metal-Chelating Properties

The complexing studies for all the unprotected hybrids were carried out through a UV–vis spectroscopy assay with the wavelength ranging from 200 to 400 nm. The unprotected hybrids were dissolved in a 5% DMSO methanolic solution (3 mM). This solution was diluted with methanol to 150 µM. 2 ml of the 150 µM solution were mixed with 2 ml of methanol to record the spectra of the hybrids alone, or with 2 ml of the salts solution (300 µM). Then, the absorption spectra of the antioxidants (75 μM) alone or in the presence of CuSO_4_, FeSO_4_, FeCl_3_ or ZnCl_2_ (150 μM) mixed for 30 min in methanol at room temperature were recorded in a 1 cm quartz cell.

### 2.4 *In Vitro* Assays

The human SH-SY5Y neuroblastoma cell line was purchased with eight passage numbers from the American Type Culture Collection (20099 Sesto San Giovanni, Milan, Italy), and maintained in Dulbecco’s modified Eagle’s medium (DMEM) (Sigma-Aldrich Ltd.) supplemented with 10% fetal bovine serum (FBS), 100 U/ml penicillin, 100 μg/ml streptomycin, into a humidified 5% CO_2_ atmosphere at 37 °C). Before carrying out the treatments, the neurons were differentiated using 10 μM of all-trans retinoid acid (Sigma Aldrich, 20151 Milan, Italy) for 5 days and maintaining a concentration of FBS at 1%. Following differentiation, when the cell lines reached a 70% confluence they have been suitably plated to carry out biologic tests.

#### 2.4.1 MTT Assay

The MTT test is based on the observation that live cells can reduce the water-soluble yellow tetrazolium salt [3-(4,5-dimethylthiazol-2-yl)-2,5-diphenyltetrazolium bromide] into water-insoluble blue/magenta formazan crystals, by dehydrogenase of the active mitochondria. Dissolved formazan crystals can be quantified using a spectrophotometer and the values obtained are in direct correlation to the number of metabolically active cells. For this reason, this colorimetric assay is used to evaluate cell viability.

The MTT assay was performed as reported in literature (Corasaniti et al., 2007). SH-SY5Y were placed in 96-well microplates at a density of 6 × 10^3^ and, the next day, were treated with the donepezil hybrids for 24 h. Subsequently, the medium was replaced with a phenol red-free medium containing MTT solution (0.5 mg/ml) and, after 4 h incubation, 100 μl of 10% SDS was added to each well to solubilize the formazan crystals.

The optical density was measured at wavelengths of 540 and 690 nm using a spectrophotometer (X MARK Spectrophotometer Microplate Bio-Rad).

In the last experiment, SH-SY5Y cells were treated with donepezil hybrids for 21 h at the concentrations shown in the Figure 5C. After that, the cells were exposed to H_2_O_2_ (100 μM, 3 h), and cell viability was measured through the MTT assay as mentioned above.

#### 2.4.2 Measurement of Reactive Oxygen Species

H2DCF-DA is a molecule that easily diffuses into cells and, through intracellular esterases, is split into H2DCF following the loss of the acetate group. H2DCF remains trapped within cells and is oxidized by intracellular ROS to form the highly fluorescent DCF compound. Spectrofluorimetric quantification of the DCF probe, provides the content of the ROS in the cell.

SH-SY5Y cells were plated in 96-well microplates at a density of 6 × 10^4^ and, the following day, were treated with donepezil hybrids for 24 h. At the end of the treatment period, to ensure that the compounds used were not degraded, cells were exposed to fresh donepezil hybrids for an additional 3 hours. Subsequently, the growth medium was replaced with a fresh phenol red-free medium containing H2DCF-DA (25 μM). After 30 min at 37°C, the cells were washed twice to remove the extracellular H2DCF-DA, centrifuged, re-suspended in PBS, exposed to H_2_O_2_ (100 μM, 30 min) and the fluorescence was evaluated by flow cytometric analysis using a FACS Accury laser flow cytometer (Becton Dickinson). The results were expressed by setting the control equal to 1 and reporting all other values. H_2_O_2_ alone was used as a positive control.

### 2.5 Prediction of Pharmacokinetic Properties

To analyze the drug like properties of the new hybrids, the prediction of the main pharmacokinetic properties was performed in silico. A comparison with hydroxytyrosol and donepezil was performed, too. Two free softwares “ADMETlab 2.0” supported by Xiangya School of Pharmaceutical Sciences, Central South University ADMETlab 2.0 (2021) https://admetmesh.scbdd.com, and “admetSAR 2.0” supported by the School of Pharmacy, East China University of Science and Technology admetSAR 2.0 (019) http://lmmd.ecust.edu.cn/admetsar2/ were used. The main properties considered were the lipo-hydrophilic character (log*P*), the permeability after oral administration (Caco-2 cell permeability), and blood-brain barrier (BBB) penetration. Finally, the agreement with Lipinski’s rules was evaluated for all compounds.

## 3 Results

### 3.1 Chemistry

**FIGURE 2:**
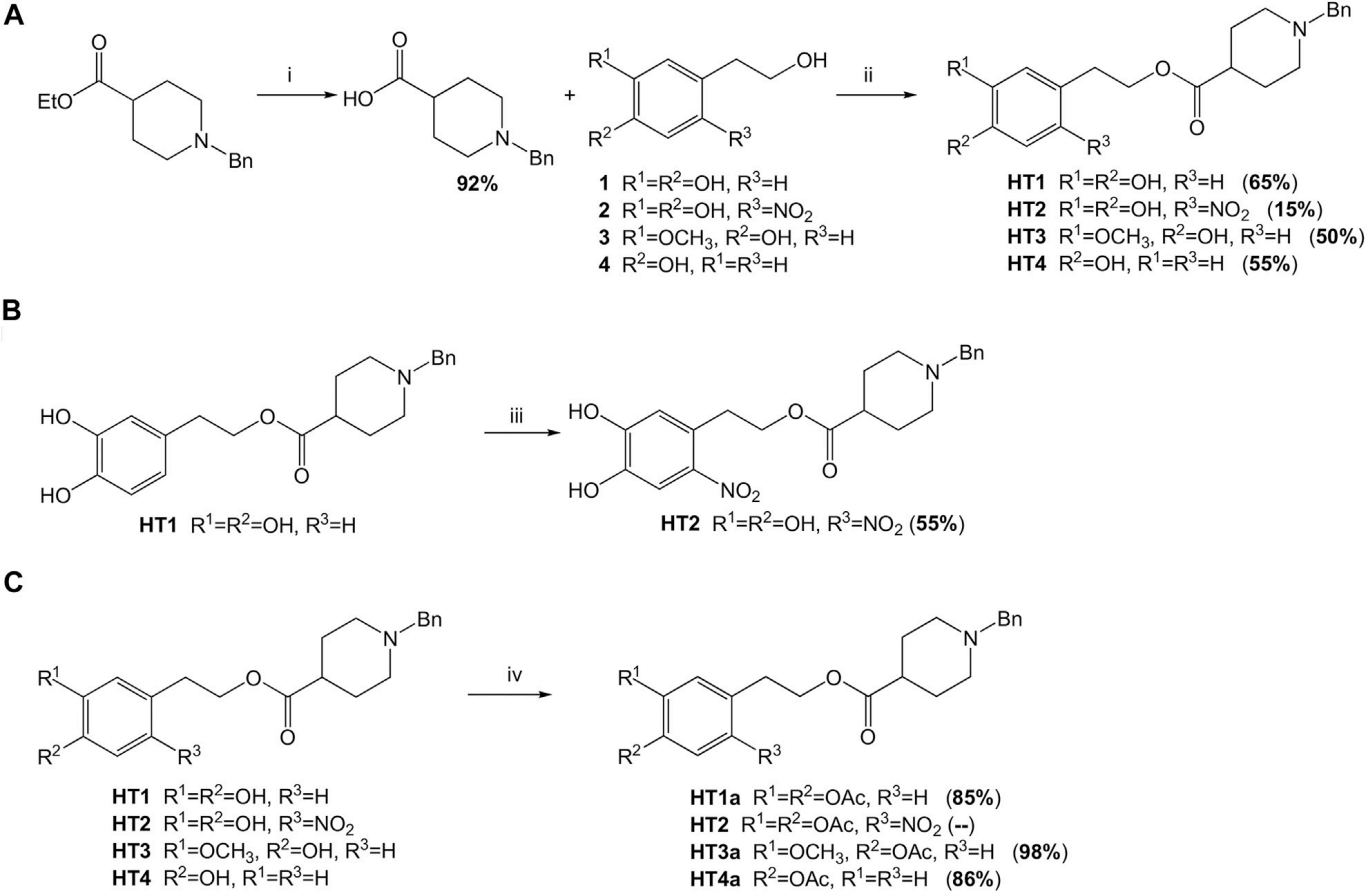
Schematic synthesis of the donepezil hybrids. **(A)** Reagents and conditions: i) 1) NaOH (0.2 N), CH_2_Cl_2_-MeOH (9:1), 48 h; 2) HCl (pH = 2–3); ii) CTMS, THF, 40°C, 6 days. **(B)** Reagents and conditions: iii) NaNO_2_, AcOH/AcONa buffer (pH = 3.75), rt, dark, 30 min **(C)** Reagents and conditions: iv) Ac_2_O dry, 4 Å MS, MW, 100°C, 25 min.

It is thought to combine the hydroxytyrosol in the donepezil structure, in place of the indanone moiety, to obtain new hybrids with potential antioxidant and metal-chelating activity. First, considering the reactivity and sensitivity of the catechol moiety of the hydroxytyrosol (HT), we worked with the acetonide precursor (Supplementary Figure S1) of the HT in a direct oxidative condensation (Mori and Togo, 2005) and in different eco-friendly transesterification reactions (Remme et al., 2007; Climent at al., 2010), but only the Steglich esterification method has given us the hybrid in good yields (English and Williams, 2009). Unfortunately, the product was disrupted during the subsequent deprotection step. Then, we tried to use the HT directly in an old esterification reaction (Brook and Chan, 1983), as reported in Figure 2A. The HT was esterified with the *N*-benzylpiperidine-4-carboxylic acid using trimethylsilyl chloride as a reagent. This allows us to esterify the HT only on the primary alcohol and not on the phenolic -OH. This method was more advantageous due to an easy operation, mild reaction conditions, simple workup and good yields. The procedure was developed considering the chemical-physical characteristics of the selected alcohols and optimized by taking the use of non-toxic solvents into account, as possible (See Table 1). The best reaction conditions were founded working at 40°C in THF for 6 days (entry 3, Table 1). This protocol was applied to the other selected polyphenolic alcohols: nitro-HT (**2**), homovanillyl alcohol (**3**), and tyrosol (**4**). Only for the 2-nitro-HT (**2**) the reaction yield was low. For this reason, we decided to perform nitration reaction (Trujillo et al., 2014) directly on the hybrid **HT1**, with a yield of 55% (Figure 2B). After that, in order to enhance the brain barrier permeation and the bioavailability, we carried out the acetylation of the phenolic groups using a green MW-assisted methodology, previously developed in our laboratory (Figure 2C) (Oliverio et al., 2016). The yields for this reaction were excellent, except for the nitro-compound. In fact, the acetylated nitro hybrid, rapidly hydrolyzed the protecting group to obtain the starting unprotected analogue. Although the compound was detected by high-resolution mass spectrometry (HRMS) in the reaction crude (Supplementary Figure S2), it was not isolated in a pure form. All compounds were purified by column chromatography. The structures were verified by ^1^H-NMR, ^13^C-NMR and HRMS. The spectra and the chemical characterization can be found in the Supplementary Material.

**TABLE 1:** Optimization of the reaction condition and synthesis of donepezil hybrids using chlorotrimethylsilane as reagent.

Entry	Alcohol	Solvent	Temp. (°C)	Time (days)	Alcohol (eq.)	CTMS (eq.)	Yield (%)^a^
1	1	DCM	r.t.	2	10	2.5	30
2	1	THF	r.t.	6	10	2.5	42
3	1	THF	40°C	6	10	2.5	65
4	1	THF	40°C	6	5	3.5	40
5	1	THF	Reflux	2	3.4	7.8	27
6	1	THF	Reflux	3	3.4	7.8	36
7	2	THF	40°C	6	10	2.5	15
8	3	THF	40°C	6	10	2.5	50
9	4	THF	40°C	6	10	2.5	55

a Isolated yields.

### 3.2 Metal-Chelating Properties

**FIGURE 3:**
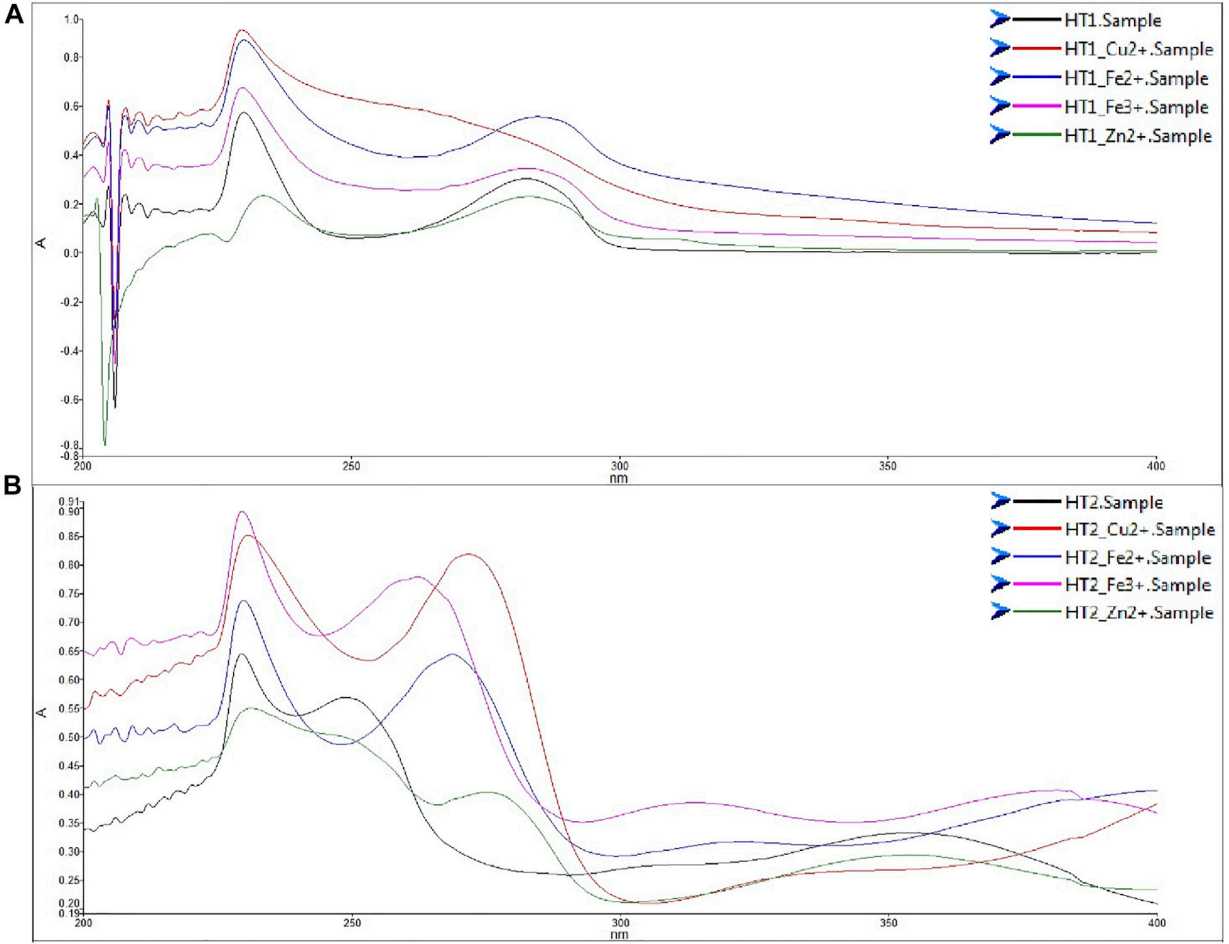
Metal chelating activity of compounds **HT1 (A)** and **HT2 (B)**. UV spectra of compounds **HT1** and **HT2** UV-vis (200–400 nm) absorption spectra of HT1 and HT2 (75 μM) alone or with CuSO_4_ (150 μM), FeSO_4_ (150 μM), FeCl_3_ (150 μM), or ZnCl_2_ (150 μM), in MeOH.

In the last decades, bio-metal dyshomeostasis in the brain was associated with both AD and PD (Morris and Levenson, 2017; Squitti et al., 2017; Ashraf et al., 2018). In particular, dysregulation of Cu^2+^, Zn^2+^, Fe^2+^ and Fe^3+^ can contribute to the ROS generation via the Fenton and Habere Weiss reaction. Then, in light of the bio-metal hypothesis of the AD pathogenesis, we tested the unprotected phenolic hybrids on the chelation properties of these redox-active metals. Since acetylation masks the key reactive portion of the phenols structure, and considering the transient nature of the acetylation as chemical modification enhancing the cellular intake, we decided to perform the *in vitro* chemical test on the unprotected derivatives. As shown in Figure 3A, the compound **HT1**, derived from HT was able to chelate only Copper (II) efficiently. Instead, the compound **HT2**, i.e., the nitro-HT hybrid, chelates all the metal cations (Figure 3B).

### 3.3 *In Vitro* Antioxidant Activities

#### 3.3.1 Oxygen Radical Absorbance Capacity by Fluorescence

The ORAC test for the unprotected hybrids was performed to evaluate the scavenging activity of samples against radical species. HT alone was also analyzed for comparison. The oxygen-radical absorbance capacity measured by fluorescence is a method that evaluates the antioxidant activity through a hydrogen atom transfer (HAT) mechanism. The results reported in Table 2 demonstrate that all compounds are more active than Trolox, a synthetic hydrophilic vitamin E analogue used as the reference compound. In particular, compound **HT1** (entry 1, Table 2) is less active than **HT** (entry 8, Table 2), the derived alcohol, while the presence of a nitro group in **HT2** reduced the antioxidant capacity by almost 3 times (entry 3, Table 2) compared to **HT1**. It is worth noting that the higher value of the Trolox equivalent was founded for the compound **HT3**, with the homovanillyl synthon in the structure (entry 4, Table 2).

**TABLE 2:** Antioxidant activity of donepezil hybrids: effects of donepezil hybrids against ROS production induced by H_2_O_2_ in SH-SY5Y cell cultures; ORAC and CUPRAC assay.

Entry	Compound	ROS measurement^a^						ORAC^b^	CUPRAC^c^
		CTRL	H_2_O_2_ (100 μM)	250 nM	500 nM	1 μM	10 μM		
1	HT1	1	6.31	6.18	5.25	5.47	5.49	21.2 ± 1.6	3.36
2	HT1a	1	6.31	2.99	2.63	2.51	2.66	n.t.^d^	n.t.^d^
3	HT2	1	6.31	2.21	1.97	1.12	1.38	7.6 ± 0.2	2.77
4	HT3	1	6.31	3.68	2.91	2.18	2.76	45.2 ± 3.6	1.80
5	HT3a	1	6.31	2.27	1.94	1.58	2.13	n.t.^d^	n.t.^d^
6	HT4	1	6.31	3.9	3.2	3.64	3.67	23.0 ± 2.0	0.99
7	HT4a	1	6.31	2.34	2.77	2.94	3.01	n.t.^d^	n.t.^d^
8	HT	n.t.	n.t.	n.t.	n.t.	n.t.	n.t.	38.8 ± 4.4	2.82
9	Trolox	n.t	n.t.	n.t.	n.t.	n.t.	n.t.	—	1

a The fluorescence values (Fitch) obtained from cytometric analysis were processed and the related quantification was conducted by setting the value of the control to 1 and comparing all other values.

b Data expressed as means ± SEM of three independent observations (µmol trolox l^−1^).

c Data are expressed as (mmol of trolox)/(mmol of tested compound).

d n.t. = not tested.

#### 3.3.2 Cupric Reducing Antioxidant Capacity Assay

An evaluation of the reducing activity for the unprotected compounds was also performed. The cupric reducing antioxidant capacity test is defined as an electron transfer (ET)-based assay for the evaluation of antioxidant action. As for the ORAC test, the antioxidant capacity of the tested hybrids was experimentally measured as Trolox equivalents, and HT was also analysed for comparison. As shown in Table 2, all hybrids demonstrated antioxidant activities comparable or higher than Trolox. **HT1** was the more active compound (entry 1, Table 2), showing an antioxidant capacity higher than **HT** on its own (entry 8, Table 2). Otherwise, as shown in the ORAC test, the reduction in the antioxidant capacity for the nitro hybrid **HT2** (entry 3, Table 2) is less pronounced, and its activity is almost high. Among the hybrids, the derivative **HT4** possesses the lower antioxidant activity (entry 6, Table 2), although it is comparable with Trolox.

### 3.4 Biological Tests

In order to evaluate the activity of donepezil hybrids on a biological level, we tested their role on human neurons SH-SY5Y, a neuroblastoma cell line widely used to study the neurotoxicity *in vitro*. First, we have assessed the impact of these compounds on cell viability in order to know their potential toxicity. Subsequently, since most neurodegenerative diseases have oxidative damage as common denominator, we wanted to study if donepezil hybrids had an antioxidant effect.

#### 3.4.1 Assessment of Cell Viability

**FIGURE 4:**
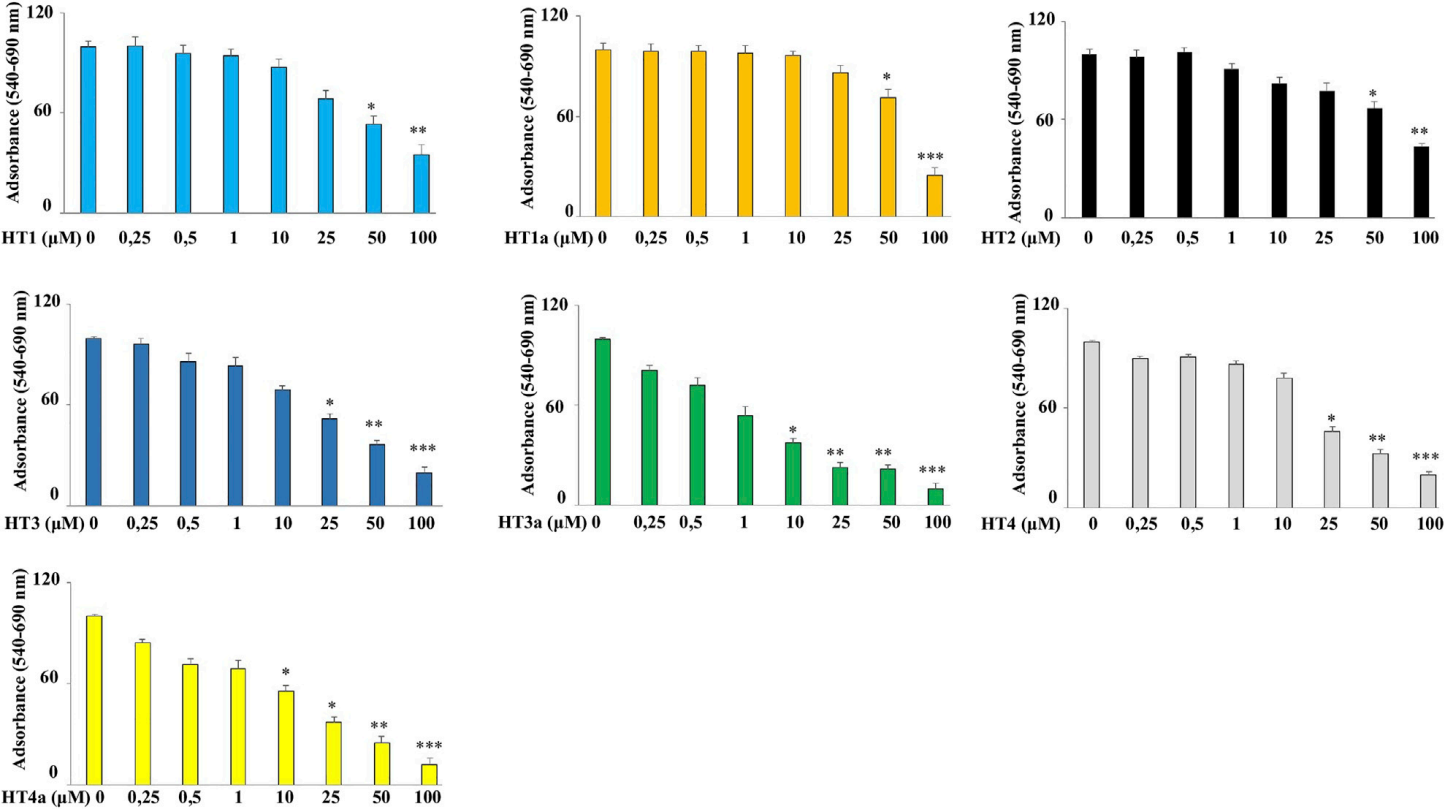
Effects of donepezil hybrids on cell viability in human neuroblastoma cells. Human neuroblastoma (SH-SY5Y) cell line was treated with donepezil hybrids for 24 h at the concentrations shown in the figure. The cell viability was measured through the MTT assay. The rate of viability of untreated cells was set at 100 and all other values were related to it. Three independent experiments were carried out, with values expressed as the mean ± standard deviation. *p < 0.05 vs. control; **p < 0.01 vs. control; ***p < 0.001 vs. control. Analysis of Variance (ANOVA) was followed by a Tukey–Kramer comparison test.

The human neuroblastoma cell line SH-SY5Y was exposed to increasing concentrations of these compounds (0.25–100 μM) for 24 h and, under these experimental conditions, all hybrids showed negligible cell death under 1 μM (Figure 4). Furthermore, data showed that all compounds are not toxic in the 1–10 μM range, and are stable in these experimental conditions. In particular, a reduction in cell viability was found for compounds **HT3a** and **HT4a** at concentrations higher than 10 μM, probably due to the higher permeability of the acetylated form.

#### 3.4.2 Measurement of Reactive Oxygen Species and Neuroprotection

**FIGURE 5:**
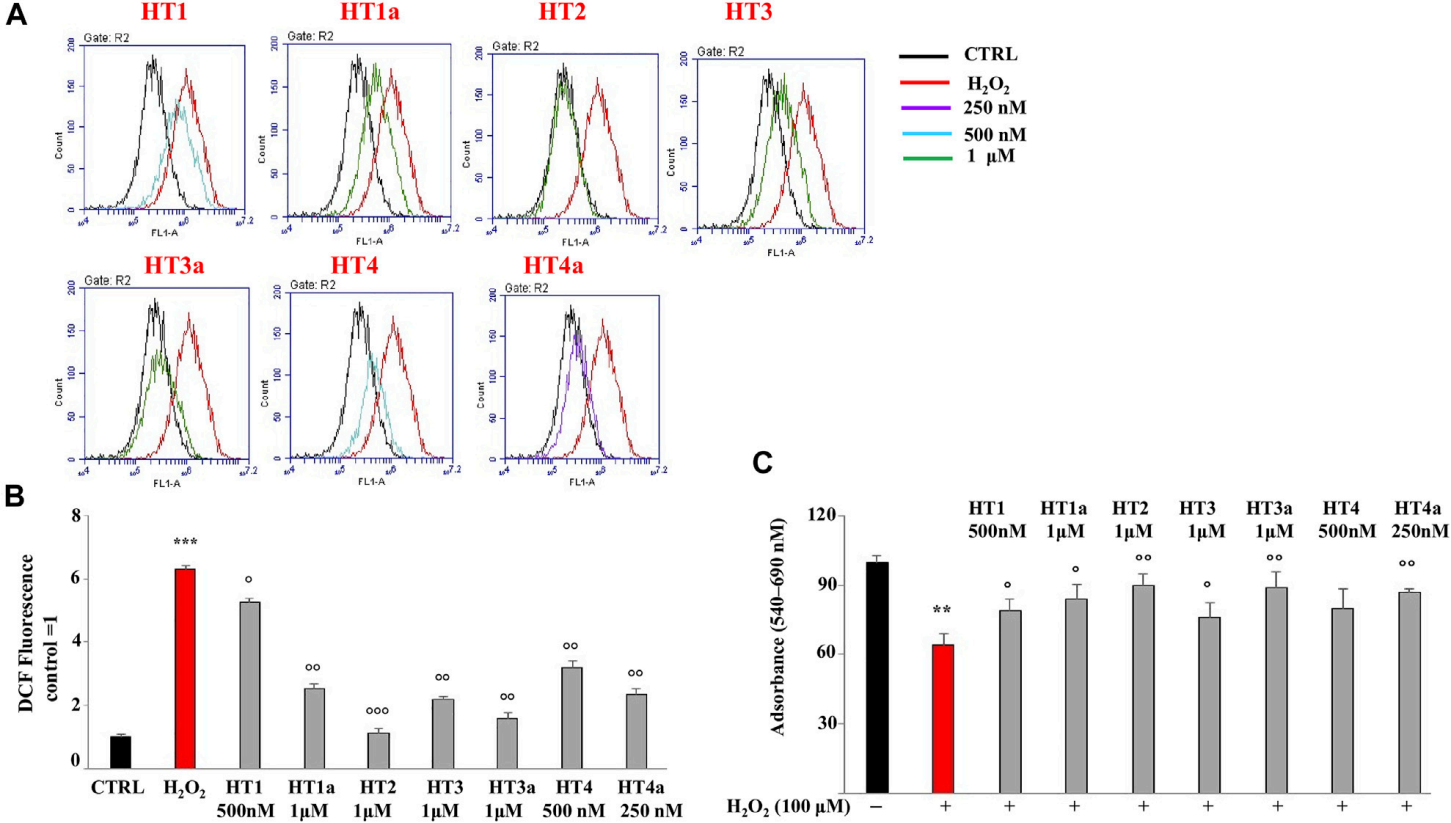
Evaluation of ROS accumulation. **(A)** Protective effects of target compounds concentration against H_2_O_2_-induced injury in SH-SY5Y cells. The most effective concentrations were chosen. The results obtained from the cytometric analysis were displayed as overlays of the individual plots. **(B**) Relative quantification of ROS accumulation. The control value was arbitrarily set to 1 and all other values were related to it. A representative result of three independent experiments (which reported the same results) is shown. Values are expressed as the mean ± standard deviation. ***p < 0.001 vs. the control. °*p* < 0.05 vs. hydrogen peroxide; °°*p* < 0.01 vs. hydrogen peroxide; °°°*p* < 0.001 vs hydrogen peroxide. Analysis of Variance (ANOVA) was followed by a Tukey–Kramer comparison test. (**C**) Effects of donepezil hybrids on damage induced by treatment with H_2_O_2_. Each value is the mean ± standard deviation of eight wells per experimental group. Results were confirmed by three independent experiments. **p < 0.01 vs. control; °*p* < 0.05 vs. hydrogen peroxide; °°*p* < 0.01 vs hydrogen peroxide. Analysis of Variance (ANOVA) was followed by a Tukey–Kramer comparison test.

Preliminary experiments were carried out in order to evaluate if donepezil hybrids had pro-oxidant effects. For this reason, SH-SY5Y cells were treated with the indicated compounds for 24 h and endogenous ROS were measured. H_2_O_2_ was used as positive control. As can be seen in Supplementary Figure S3, no compound has resulted in an accumulation of ROS at the indicated concentrations (0.25–100 μM). Subsequently, the antioxidant properties of the compounds were evaluated and conditions of oxidative stress in neuroblastoma cells were simulated by using H_2_O_2_ as toxic insult. The treatment time, with H_2_O_2_ used to produce an accumulation of ROS (30 min), did not cause reduction in cell viability. In order to obtain a significant reduction in viability we need to treat SH-SY5Y cells for 3 hours or more (see Supplementary Figure S4). Thus, exposure to H_2_O_2_ for 30 min causes only oxidative damage. In particular, Figures 5A,B shows the protective effect against H_2_O_2_, and among all concentrations used, those with the most protective effects were highlighted. The ROS values corresponding to the other concentrations are summarised in Table 2. The nitro hybrid **HT2** was the more effective one to reduce the ROS amount to the physiological values. Furthermore, it is noteworthy that for the compounds **HT3**, the higher antioxidant effect it was measured in the acetylated form (**HT3a**). Then, the IC_50_ values are reported in Table 3, together with the potential therapeutic index of these hybrids (expressed by the ratio between the concentration of the hybrid that causes a toxic effect and the concentration that causes an antioxidant effect in neuronal cells). As shown in Table 3, all hybrids possess a neurotoxic concentration higher than the concentration showing antioxidant activity. Compound **HT1** showed the best therapeutic index ratio, while for compound **HT3a** the lower value was calculated, although it is equal to 10. Finally, to confirm the promising results obtained with the evaluation of antioxidant activity, we investigated whether donepezil hybrids were able to reduce the proliferative damage induced by exposure to H_2_O_2_. As shown in Figure 5C the co-treatment with donepezil hybrids and H_2_O_2_ had shown a protective effect. In particular, **HT2**, **HT3a** and **HT4a** showed a statistically significant increase in cell viability compared to the effect caused by treatment with hydrogen peroxide alone.

**TABLE 3:** IC_50_ values and therapeutic index of new donepezil hybrids.

Compounds	IC_50_ (μM)	Therapeutic index^a^
HT1	50	100
HT1a	>50	50
HT2	>50	50
HT3	>25	25
HT3a	>10	10
HT4	>25	50
HT4a	>10	40

a Expressed as a ratio between the IC_50_ value and the concentration with more antioxidant activity.

### 3.5 Prediction of Pharmacokinetic Properties

A preliminary assessment of the drug-likeness of the new hybrids was performed by using two different softwares: ADMETlab 2.0 developed by Xiong et al. (2021), and admetSAR 2.0 developed by Yang et al. (2018). The octanol/water partition coefficient (logP or AlogP, respectively) was calculated as an essential parameter for *Lipinski’s rule of five*. In all cases, the calculated logP was lower than 5, and all hybrids respect *Lipinski’s rule of five* (Table 4). The expected permeability of these new compounds for the Caco-2 cell line, as a model for the absorption prediction after oral administration, and the BBB were also evaluated. On these two parameters, some discordant results were registered by the two prediction models. For the ADMETlab software, the predicted Caco-2 permeability (given as the log cm/s) is considered excellent if it has a predicted value higher than −5.15 log cm/s. Among the new hybrids, only compound **HT1** was found to have a predicted value lower than −5.15, while according to the admetSAR software, compounds **HT1**, **HT1a,** and **HT2** showed a certain probability of not being permeable. Otherwise, the prediction on the BBB penetration was calculated as highly favorable for all new hybrids, except for compounds **HT4** and **HT4a,** by the admetSAR software, while the ADMETlab prediction model has considered excellent values for **HT1, HT3**, **HT4** (range 0.7–1.0), and medium values for the others (range 0.3–0.7). All hybrids were also compared with **HT** alone and donepezil. It is worth noting that, while for compound **HT2** the Caco-2 permeability seems to be unfavorable, for one of the ADME prediction softwares (admetSAR 2.0), the same software considers it as the only one (except Donepezil) to have a potential human oral bioavailability (data not shown).

**TABLE 4:** Pharmacokinetic properties as predicted in silico by software ADMETlab 2.0 and admetSAR 2.0

Compound	MW	ADMETlab			admetSAR			Violation of Lipinskys’rule
		logP^a^	BBB penetration^b^	Caco-2 permeability^c^	AlogP^a^	BBB penetration^d^	Caco-2 permeability^d^	
HT1	355.434	3.147	0.893	−5.214	3.10	(+) 0.9128	(−) 0.5362	0
HT1a	439.508	3.128	0.361	−4.397	3.54	(+) 0.9509	(−) 0.5712	0
HT2	400.431	3.121	0.463	−5.103	3.0	(+) 0.9693	(−) 0.7994	0
HT3	369.461	3.415	0.984	−4.911	3.40	(+) 0.9366	(+) 0.6406	0
HT3a	411.498	3.347	0.667	−4.873	3.62	(+) 0.9768	(+) 0.6086	0
HT4	339.435	3.533	0.947	−4.94	3.39	(−) 0.2371	(+) 0.6969	0
HT4a	381.472	3.545	0.401	−4.872	3.61	(−) 0.3715	(+) 0.6051	0
HT	154.165	0.174	0.037	−4.433	0.63	(−) 0.5648	(+) 0.6302	0
Donepezil	379.5	4.191	0.975	−4,793	4.36	(+) 1.0000	(+) 0.6843	0

a Log of the octanol/water partition coefficient.

b The output value is the probability of being BBB+.

c Optimal: higher than −5.15 Log unit.

d Value (+) or (−) indicating the probability of being BBB(+) or BBB(−), and the permeability or not towards Caco-2 cell line, respectively.

## 4 Discussion

The importance of the redox state of the cells is crucial not only for the physiological metabolism of an organism but also in the development of several age-related, cardiovascular and neoplastic diseases (Liguori et al., 2018). Diverse authors demonstrated the neuroprotective effects of the HT derivatives against not only AD (Klimova et al., 2019) but also against PD (Palazzi et al., 2018). In light of these pieces of evidence, we tried to insert the HT into the donepezil structure to exploit its neuroprotection effect. Then, we planned to also synthesize tyrosol and homovanillyl alcohol hybrids to evaluate the influence of the *ortho*-diphenolic moiety in the tested biological activities. Noteworthy is the presence of a nitrocatechol moiety in two catechol *O*-methyltransferase (COMT) inhibitors such as tolcapone and entacapone, included in the clinical treatment of PD (Li et al., 2017; Artusi et al., 2021). Furthermore, several studies demonstrated a remarkable activity of nitro-HT derivatives in the dopamine metabolism, suggesting a putative effect against PD as novel and lipophilic COMT inhibitors (Gallardo et al., 2014; Trujillo et al., 2014). First, we tested the chelating ability of all these hybrids in their unprotected form, since the primary role of the acetyl groups is that of enhancing the analogues bioavailability. Only **HT1** and **HT2** showed metal chelating properties, highlighting the importance of the catechol moiety in the metal coordination. In particular the presence of a nitro-group allowed to chelate not only Cu(II), but also the other redox-active metal ions Fe(II), Fe(III), and Zn(II). The cell viability of these hybrids was evaluated to exclude a potential cytotoxic effect. Encouraging results were obtained for all of the hybrids, which did not show substantial cell death until 1 µM concentration. Another crucial issue, related to the employment of natural polyphenol compounds is that, in some cases, they could act as pro-oxidants causing DNA damage and mutagenesis, depending on the concentration or the cellular environment (León-González et al., 2015; Eghbaliferiz and Iranshahi, 2016). To exclude a pro-oxidant effect for these new hybrids, the SH-SY5Y neuroblastoma cell line, was treated for 24 h with them, using H_2_O_2_ as a positive control. The measurement of the endogenous ROS did not show pro-oxidant effects even at the highest concentrations for all hybrids (Supplementary Figure S3). This important result, together with the cell viability, was fundamental to assess the treatment assurance with the target compounds. Then, conditions of oxidative stress in neuroblastoma cells were simulated by using H_2_O_2_ as toxic insult. Some of these hybrids, **HT3a** and **HT2**, significantly showed protective effects against the damage induced by H_2_O_2_ at 100 µM. In particular, **HT2** showed the highest antioxidant capacity preventing the ROS formation evoked by H_2_O_2_ in SH-SY5Y cells, and restoring the ROS concentration to the physiological values. In the attempt, to elucidate the mechanism of the antioxidant activity for the synthesized compounds in the unprotected form, two different and complementary chemical tests were performed. ORAC and CUPRAC tests were selected as hydrogen atom transfer (HAT)- and electron transfer (ET)-based assays, respectively. The antioxidant activities of the unprotected compounds were provided in Trolox equivalent and comparatively studied against results obtained free HT. Surprisingly, discordant results were obtained for the two tests, in particular for the nitro-hybrids **HT2,** which seemed to be the less active in the ORAC test, although maintaining high antioxidant capacity in the CUPRAC test. On the contrary, the **HT3** was the more active in the HAT-based ORAC assay, while showing a not excellent antioxidant capacity in the electron transfer (ET)-based CUPRAC assay. Although these chemical tests only possess a predictive valence, these results suggest that both the direct scavenging capability of the homovanillyl hybrid **HT3** and the reducing capacity of the nitro hybrid **HT2** are very important for the resulting antioxidant activity. It is worth noting that for the homovanillyl hybrid the best result on the ROS reduction was obtained by the acetylated compound **HT3a**, showing the importance of cell permeability in the expression of the biological activity. Moreover, these preliminary results meet the margin of safety measured for the two hybrids through the therapeutic index, (higher for compound **HT2** than **HT3a**), allowing further investigation of these promising compounds. Neuroprotection activity against the cytotoxicity elicited by H_2_O_2_ was also assessed by measuring the cell viability using MTT assay. As already reported about the ROS reduction, hybrids **HT2** and **HT3a** were able to attenuate cell injury against oxidative stress. Finally, to assess the potential of these compounds as drugs, some pharmacokinetics properties were calculated *in silico.* Two different prediction models were employed to evaluate the agreement with *Lipinski’s rule of five*, the Caco-2 cell line permeability and the BBB penetration. For compound **HT3a,** the two different softwares predicted almost good results for all of the parameters, while discordant results were registered by the two prediction models for compound **HT2**. On one hand, the ADMETlab 2.0 software predicted medium BBB penetration and good Caco-2 cell permeability. On the other hand, the admetSAR 2.0 software considered not excellent permeation properties together with very good value for the BBB penetration. Although the pharmacokinetic properties of all the hybrids are less favorable in terms of Caco-2 permeability or BBB penetration than those of donepezil, we obtained better results with **HT2** and **HT3a,** if compared to just **HT**. Thus confirming the goal to enhance the **HT** activity towards the Central Nervous System, these encouraging results could suggest that these two hybrids could be eligible as drug candidates.

In conclusion, we report the synthesis of new biophenol-donepezil hybrids for the potential treatment of neurodegenerative diseases. These new synthetic hybrids were obtained in good yields. Chemical and biological assays were performed in order to evaluate their multi target directed ligand effects. These preliminary results on the antioxidant activity highlighted the efficacy of compounds **HT2** and **HT3a**. In particular, the homovanillyl hybrid **HT3a** possesses a good antioxidant capacity, probably through a hydrogen transfer mechanism. The higher activity measured in the cell culture for the acetylated compound confirmed the importance of the cell membrane permeation to carry out the biological activity. As for the **HT2** hybrid, bringing a nitro group onto the HT moiety, it showed interesting metal chelating properties associated with negligible cellular toxicity, as well as an electron-transfer antioxidant power. Such differentiated mechanisms of action enhance the possible biological applications of these polyphenols-donepezil hybrids, paving the way to other different targets in the neurodegenerative disorders.

## Data Availability

The original contributions presented in the study are included in the article/Supplementary Material, further inquiries can be directed to the corresponding authors.

## References

[B1] Artusi C. A. Sarro L. Imbalzano G. Fabbri M. Lopiano L. (2021). Safety and Efficacy of Tolcapone in Parkinson's Disease: Systematic Review. Eur. J. Clin. Pharmacol. 77 (6), 817–829. 10.1007/s00228-020-03081-x 33415500 PMC8128808

[B2] Ashraf A. Clark M. So P.-W. (2018). The Aging of Iron Man. Front. Aging Neurosci. 10, 65. 10.3389/fnagi.2018.00065 29593525 PMC5857593

[B3] Balakrishnan R. Azam S. Cho D.-Y. Su-Kim I. Choi D.-K. (2021). Natural Phytochemicals as Novel Therapeutic Strategies to Prevent and Treat Parkinson's Disease: Current Knowledge and Future Perspectives. Oxidative Med. Cell Longevity 2021, 1–32. 10.1155/2021/6680935 PMC8169248 34122727

[B4] Barnham K. J. Bush A. I. (2008). Metals in Alzheimer's and Parkinson's Diseases. Curr. Opin. Chem. Biol. 12 (2), 222–228. 10.1016/j.cbpa.2008.02.019 18342639

[B5] Batarseh Y. S. Kaddoumi A. (2018). Oleocanthal-rich Extra-virgin Olive Oil Enhances Donepezil Effect by Reducing Amyloid-β Load and Related Toxicity in a Mouse Model of Alzheimer's Disease. J. Nutr. Biochem. 55, 113–123. 10.1016/j.jnutbio.2017.12.006 29413486 PMC5936648

[B6] Blesa J. Trigo-Damas I. Quiroga-Varela A. Jackson-Lewis V. R. (2015). Oxidative Stress and Parkinson's Disease. Front. Neuroanat. 9, 91. 10.3389/fnana.2015.00091 26217195 PMC4495335

[B7] Brook M. A. Chan T. H. (1983). A Simple Procedure for the Esterification of Carboxylic Acids. Synthesis 1983, 201–203. 10.1055/s-1983-302791983

[B8] Bulotta S. Oliverio M. Russo D. Procopio A. (2013). “Biological Activity of Oleuropein and its Derivatives,” in Chapter 119: Biological Activity of Oleuropein and its Derivatives. Natural Products. Editors Ramawat K. G. Merillon J. M. (Berlin: Springer-Verlag Berlin Heidelberg), 3605–3638. 10.1007/978-3-642-22144-6-015610.1007/978-3-642-22144-6_156

[B9] Caliandro R. Pesaresi A. Cariati L. Procopio A. Oliverio M. Lamba D. (2018). Kinetic and Structural Studies on the Interactions of *Torpedo californica* Acetylcholinesterase with Two Donepezil-like Rigid Analogues. J. Enzyme Inhib. Med. Chem. 33, 794–803. 10.1080/14756366.2018.1458030 29651884 PMC6009889

[B10] Cariati L. Oliverio M. Mutti F. G. Bonacci S. Knaus T. Costanzo P. (2019). Hydrolases-mediated Transformation of Oleuropein into Demethyloleuropein. Bioorg. Chem. 84, 384–388. 10.1016/j.bioorg.2018.12.005 30543985

[B11] Climent M. J. Corma A. De Frutos P. Iborra S. NoyVelty M. A. Velty A. (2010). Chemicals from Biomass: Synthesis of Glycerol Carbonate by Transesterification and Carbonylation with Urea with Hydrotalcite Catalysts. The Role of Acid-Base Pairs. J. Catal. 269, 140–149. 10.1016/j.jcat.2009.11.001

[B12] Corasaniti M. T. Maiuolo J. Maida S. Fratto V. Navarra M. Russo R. (2007). Cell Signaling Pathways in the Mechanisms of Neuroprotection Afforded by Bergamot Essential Oil against NMDA-Induced Cell Deathin Vitro. Br. J. Pharmacol. 151 (4), 518–529. 10.1038/sj.bjp.0707237 17401440 PMC2013960

[B13] Costa M. Josselin R. Silva D. F. Cardoso S. M. May N. V. Chaves S. (2020). Donepezil-based Hybrids as Multifunctional Anti-alzheimer's Disease Chelating Agents: Effect of Positional Isomerization. J. Inorg. Biochem. 206, 111039. 10.1016/j.jinorgbio.2020.111039 32171933

[B14] Costanzo P. Bonacci S. Cariati L. Nardi M. Oliverio M. Procopio A. (2018). Simple and Efficient Sustainable Semi-synthesis of Oleacein [2-(3,4-hydroxyphenyl) Ethyl (3S,4E)-4-Formyl-3-(2-Oxoethyl)hex-4-Enoate] as Potential Additive for Edible Oils. Food Chem. 245, 410–414. 10.1016/j.foodchem.2017.10.097 29287389

[B15] Costanzo P. Cariati L. Desiderio D. Sgammato R. Lamberti A. Arcone R. (2016). Design, Synthesis, and Evaluation of Donepezil-like Compounds as AChE and BACE-1 Inhibitors. ACS Med. Chem. Lett. 7 (5), 470–475. 10.1021/acsmedchemlett.5b00483 27190595 PMC4867475

[B16] Crespo M. C. Tomé-Carneiro J. Pintado C. Dávalos A. Visioli F. Burgos-Ramos E. (2017). Hydroxytyrosol Restores Proper Insulin Signaling in an Astrocytic Model of Alzheimer's Disease. Biofactors 43 (4), 540–548. 10.1002/biof.1356 28317262

[B17] Cui X. Lin Q. Liang Y. (2020). Plant-Derived Antioxidants Protect the Nervous System from Aging by Inhibiting Oxidative Stress. Front. Aging Neurosci. 12, 209. 10.3389/fnagi.2020.00209 32760268 PMC7372124

[B18] Cuyàs E. Verdura S. Lozano-Sánchez J. Viciano I. Llorach-Parés L. Nonell-Canals A. (2019). The Extra virgin Olive Oil Phenolic Oleacein Is a Dual Substrate-Inhibitor of Catechol-O-Methyltransferase. Food Chem. Toxicol. 128, 35–45. 10.1016/j.fct.2019.03.049 30935952

[B19] Dias K. S. T. de Paula C. T. Dos Santos T. Souza I. N. O. Boni M. S. Guimarães M. J. R. (2017). Design, Synthesis and Evaluation of Novel Feruloyl-Donepezil Hybrids as Potential Multitarget Drugs for the Treatment of Alzheimer's Disease. Eur. J. Med. Chem. 130, 440–457. 10.1016/j.ejmech.2017.02.043 28282613

[B20] Dinda B. Dinda M. Kulsi G. Chakraborty A. Dinda S. (2019). Therapeutic Potentials of Plant Iridoids in Alzheimer's and Parkinson's Diseases: A Review. Eur. J. Med. Chem. 169, 185–199. 10.1016/j.ejmech.2019.03.009 30877973

[B21] Eghbaliferiz S. Iranshahi M. (2016). Prooxidant Activity of Polyphenols, Flavonoids, Anthocyanins and Carotenoids: Updated Review of Mechanisms and Catalyzing Metals. Phytother. Res. 30 (9), 1379–1391. 10.1002/ptr.5643 27241122

[B22] English B. J. Williams R. M. (2009). Synthesis of (±)-oleocanthal via a Tandem Intramolecular Michael Cyclization-HWE Olefination. Tetrahedron Lett. 50, 2713–2715. 10.1016/j.tetlet.2009.03.145 20161637 PMC2817976

[B23] Gallardo E. Madrona A. Palma-Valdés R. Trujillo M. Espartero J. L. Santiago M. (2014). The Effect of Hydroxytyrosol and its Nitroderivatives on Catechol-*O*-Methyl Transferase Activity in Rat Striatal Tissue. RSC Adv. 4, 61086–61091. 10.1039/C4RA09872B

[B24] Gambacorta A. Tofani D. Bernini R. Migliorini A. (2007). High-Yielding Preparation of a Stable Precursor of Hydroxytyrosol by Total Synthesis and from the Natural Glycoside Oleuropein. J. Agric. Food Chem. 55 (9), 3386–3391. 10.1021/jf063353b 17411065

[B25] Ganguly U. Kaur U. Chakrabarti S. S. Sharma P. Agrawal B. K. Saso L. (2021). Oxidative Stress, Neuroinflammation, and NADPH Oxidase: Implications in the Pathogenesis and Treatment of Alzheimer's Disease. Oxidative Med. Cell Longevity 2021, 1–19. 10.1155/2021/7086512 PMC8068554 33953837

[B26] Gardener H. Caunca M. R. (2018). Mediterranean Diet in Preventing Neurodegenerative Diseases. Curr. Nutr. Rep. 7 (1), 10–20. 10.1007/s13668-018-0222-5 29892785 PMC7212497

[B27] Khalatbary A. R. (2013). Olive Oil Phenols and Neuroprotection. Nutr. Neurosci. 16 (6), 243–249. 10.1179/1476830513Y.0000000052 23406576

[B28] Kim G. H. Kim J. E. Rhie S. J. Yoon S. (2015). The Role of Oxidative Stress in Neurodegenerative Diseases. Exp. Neurobiol. 24 (4), 325–340. 10.5607/en.2015.24.4.325 26713080 PMC4688332

[B29] Klimova B. Novotný M. Kuca K. Valis M. (2019). Effect of an Extra-Virgin Olive Oil Intake on the Delay of Cognitive Decline: Role of Secoiridoid Oleuropein? Ndt Vol. 15, 3033–3040. 10.2147/NDT.S218238 PMC6825477 31754302

[B30] León-González A. J. Auger C. Schini-Kerth V. B. (2015). Pro-oxidant Activity of Polyphenols and its Implication on Cancer Chemoprevention and Chemotherapy. Biochem. Pharmacol. 98 (3), 371–380. 10.1016/j.bcp.2015.07.017 26206193

[B31] Leri M. Oropesa-Nuñez R. Canale C. Raimondi S. Giorgetti S. Bruzzone E. (2018). Oleuropein Aglycone: A Polyphenol with Different Targets against Amyloid Toxicity. Biochim. Biophys. Acta (Bba) - Gen. Subjects 1862 (6), 1432–1442. 10.1016/j.bbagen.2018.03.023 29571746

[B32] Li J. Lou Z. Liu X. Sun Y. Chen J. (2017). Efficacy and Safety of Adjuvant Treatment with Entacapone in Advanced Parkinson's Disease with Motor Fluctuation: A Systematic Meta-Analysis. Eur. Neurol. 78 (3-4), 143–153. 10.1159/000479555 28813703

[B33] Li Q. He S. Chen Y. Feng F. Qu W. Sun H. (2018). Donepezil-based Multi-Functional Cholinesterase Inhibitors for Treatment of Alzheimer's Disease. Eur. J. Med. Chem. 158, 463–477. 10.1016/j.ejmech.2018.09.031 30243151

[B34] Liguori I. Russo G. Curcio F. Bulli G. Aran L. Della-Morte D. (2018). Oxidative Stress, Aging, and Diseases. Cia Vol. 13, 757–772. 10.2147/CIA.S158513 PMC5927356 29731617

[B35] Maiuolo L. Feriotto G. Algieri V. Nardi M. Russo B. Di Gioia M. L. (2017). Antiproliferative Activity of Novel Isatinyl/indanyl Nitrones (INs) as Potential Spin Trapping Agents of Free Radical Intermediates. Med. Chem. Commun. 9 (2), 299–304. 10.1039/c7md00537g PMC6083742 30108923

[B36] Mori N. Togo H. (2005). Facile Oxidative Conversion of Alcohols to Esters Using Molecular Iodine. Tetrahedron 61, 5915–5925. 10.1016/j.tet.2005.03.097

[B37] Morris D. R. Levenson C. W. (2017). Neurotoxicity of Zinc. Neurotoxicity of Metals. Springer, 303–312. 10.1007/978-3-319-60189-2_15 28889274

[B38] Nardi M. Bonacci S. Cariati L. Costanzo P. Oliverio M. Sindona G. (2017). Synthesis and Antioxidant Evaluation of Lipophilic Oleuropein Aglycone Derivatives. Food Funct. 8 (12), 4684–4692. 10.1039/c7fo01105a 29160876

[B39] Neha K. Haider M. R. Pathak A. Yar M. S. (2019). Medicinal Prospects of Antioxidants: A Review. Eur. J. Med. Chem. 178, 687–704. 10.1016/j.ejmech.2019.06.010 31228811

[B40] Oliverio M. Costanzo P. Nardi M. Calandruccio C. Salerno R. Procopio A. (2016). Tunable Microwave-Assisted Method for the Solvent-free and Catalyst-free Peracetylation of Natural Products. Beilstein J. Org. Chem. 12, 2222–2233. 10.3762/bjoc.12.214 27829931 PMC5082547

[B41] Oliverio M. Nardi M. Cariati L. Vitale E. Bonacci S. Procopio A. (2016). "On Water" MW-Assisted Synthesis of Hydroxytyrosol Fatty Esters. ACS Sust. Chem. Eng. 4, 661–665. 10.1021/acssuschemeng.5b01201

[B42] Oliverio M. Nardi M. Di Gioia M. L. Costanzo P. Bonacci S. Mancuso S. (2021). Semi-synthesis as a Tool for Broadening the Health Applications of Bioactive Olive Secoiridoids: a Critical Review. Nat. Prod. Rep. 38, 444–469. 10.1039/D0NP00084A 33300916

[B43] Ou B. Hampsch-Woodill M. Prior R. L. (2001). Development and validation of an improved oxygen radical absorbance capacity assay using fluorescein as the fluorescent probe, J. Agric. Food Chem. 49, 4619–4626. 10.1021/jf010586o 11599998

[B44] Ozyürek M. Bektaşoğlu B. Güçlü K. Güngör N. Apak R. (2008). Simultaneous Total Antioxidant Capacity Assay of Lipophilic and Hydrophilic Antioxidants in the Same Acetone-Water Solution Containing 2% Methyl-Beta-Cyclodextrin Using the Cupric Reducing Antioxidant Capacity (CUPRAC) Method. Anal. Chim. Acta 630 (1), 28–39. 10.1016/j.aca.2008.09.057 19068323

[B45] Palazzi L. Bruzzone E. Bisello G. Leri M. Stefani M. Bucciantini M. (2018). Oleuropein Aglycone Stabilizes the Monomeric α-synuclein and Favours the Growth of Non-toxic Aggregates. Sci. Rep. 8 (1), 8337. 10.1038/s41598-018-26645-5 29844450 PMC5974307

[B46] Schneider C. Remme N. Koschek K. (2007). Scandium Triflate Catalyzed Transesterification of Carboxylic Esters. Synlett 2007, 0491–0493. 10.1055/s-2007-967936

[B47] Singh A. Kukreti R. Saso L. Kukreti S. (2019). Oxidative Stress: A Key Modulator in Neurodegenerative Diseases. Molecules 24 (8), 1583. 10.3390/molecules24081583 PMC6514564 31013638

[B48] Spagnuolo C. Napolitano M. Tedesco I. Moccia S. Milito A. Luigi Russo G. (2016). Neuroprotective Role of Natural Polyphenols. Ctmc 16 (17), 1943–1950. 10.2174/1568026616666160204122449 26845551

[B49] Squitti R. Ventriglia M. Siotto M. Salustri C. (2017). Copper in Alzheimer's Disease. Biometals in Neurodegenerative Diseases. Amsterdam. Elsevier, 19–34. 10.1016/b978-0-12-804562-6.00002-6

[B50] St-Laurent-Thibault C. Arseneault M. Longpré F. Ramassamy C. (2011). Tyrosol and Hydroxytyrosol Two Main Components of Olive Oil, Protect N2a Cells against Amyloid-β-Induced Toxicity. Involvement of the NF-Κb Signaling. Car 8 (5), 543–551. 10.2174/156720511796391845 21605049

[B51] Theodorou V. Skobridis K. Tzakos A. G. Ragoussis V. (2007). A Simple Method for the Alkaline Hydrolysis of Esters. Tetrahedron Lett. 48, 8230–8233. 10.1016/j.tetlet.2007.09.074

[B52] Trujillo M. Gallardo E. Madrona A. Bravo L. Sarriá B. González-Correa J. A. (2014). Synthesis and Antioxidant Activity of Nitrohydroxytyrosol and its Acyl Derivatives. J. Agric. Food Chem. 62 (42), 10297–10303. 10.1021/jf503543x 25264851

[B53] Unzeta M. Esteban G. Bolea I. Fogel W. A. Ramsay R. R. Youdim M. B. H. (2016). Multi-Target Directed Donepezil-like Ligands for Alzheimer's Disease. Front. Neurosci. 10, 205. 10.3389/fnins.2016.00205 27252617 PMC4879129

[B54] Xiong G. Wu Z. Yi J. Fu L. Yang Z. Hsieh C. (2021). ADMETlab 2.0: an Integrated Online Platform for Accurate and Comprehensive Predictions of ADMET Properties. Nucleic Acids Res., 49, W5–W14. https://admetmesh.scbdd.com/. 33893803 10.1093/nar/gkab255 PMC8262709

[B55] Xu W. Wang X.-B. Wang Z.-M. Wu J.-J. Li F. Wang J. (2016). Synthesis and Evaluation of Donepezil-Ferulic Acid Hybrids as Multi-Target-Directed Ligands against Alzheimer's Disease. Med. Chem. Commun. 7, 990–998. 10.1039/C6MD00053C

[B56] Yang H. Lou C. Sun L. Li J. Cai Y. Wang Z. (2019). admetSAR 2.0: Web-Service for Prediction and Optimization of Chemical ADMET Properties. Bioinformatics. 35 (6), 1067–1069. http://lmmd.ecust.edu.cn/admetsar2/. 30165565 10.1093/bioinformatics/bty707

[B57] Yang H. Lou C. Sun L. Li J. Cai Y. Wang Z. (2018). admetSAR 2.0: Web-Service for Prediction and Optimization of Chemical ADMET Properties. Bioinformatics 35 (6), 1067–1069. 10.1093/bioinformatics/bty707 30165565

