# Supplementary material for: Novel Hydroxytyrosol-Donepezil Hybrids as Potential Antioxidant and Neuroprotective Agents
Source: Front Chem. 2021 Oct 19;9:741444. doi: 10.3389/fchem.2021.741444 (PMC8560896; doi:10.3389/fchem.2021.741444)
Supplement: Supplementary file 1 [file DataSheet1.pdf]

Supplementary Material

1 Synthetic pathways tested for the synthesis of the hydroxytyrosol-donepezil hybrid

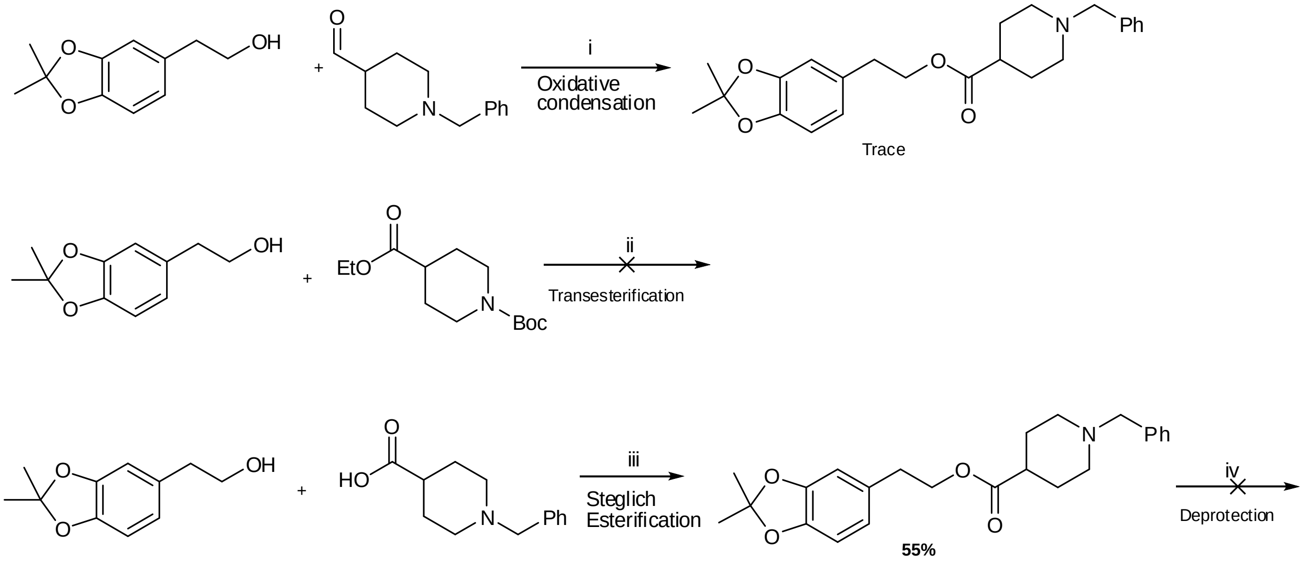

Supplementary Figure 1. Reaction conditions: (i) I_2, K_2CO_3, *t*-BuOH, rt, N_2, 4 days; (ii) $Sc(OTf)_3$, MW, 160 °C, 40 min; or MgO, 70 °C, N_2, o.n.; (iii) DIC, DMAP, CH_2Cl_2 dry / DMF dry (1:1), 0 °C to rt, o.n., N_2; (iv) Amberlist, MeOH, reflux.

2 LC-HRMS identification of HT2 acetylation products

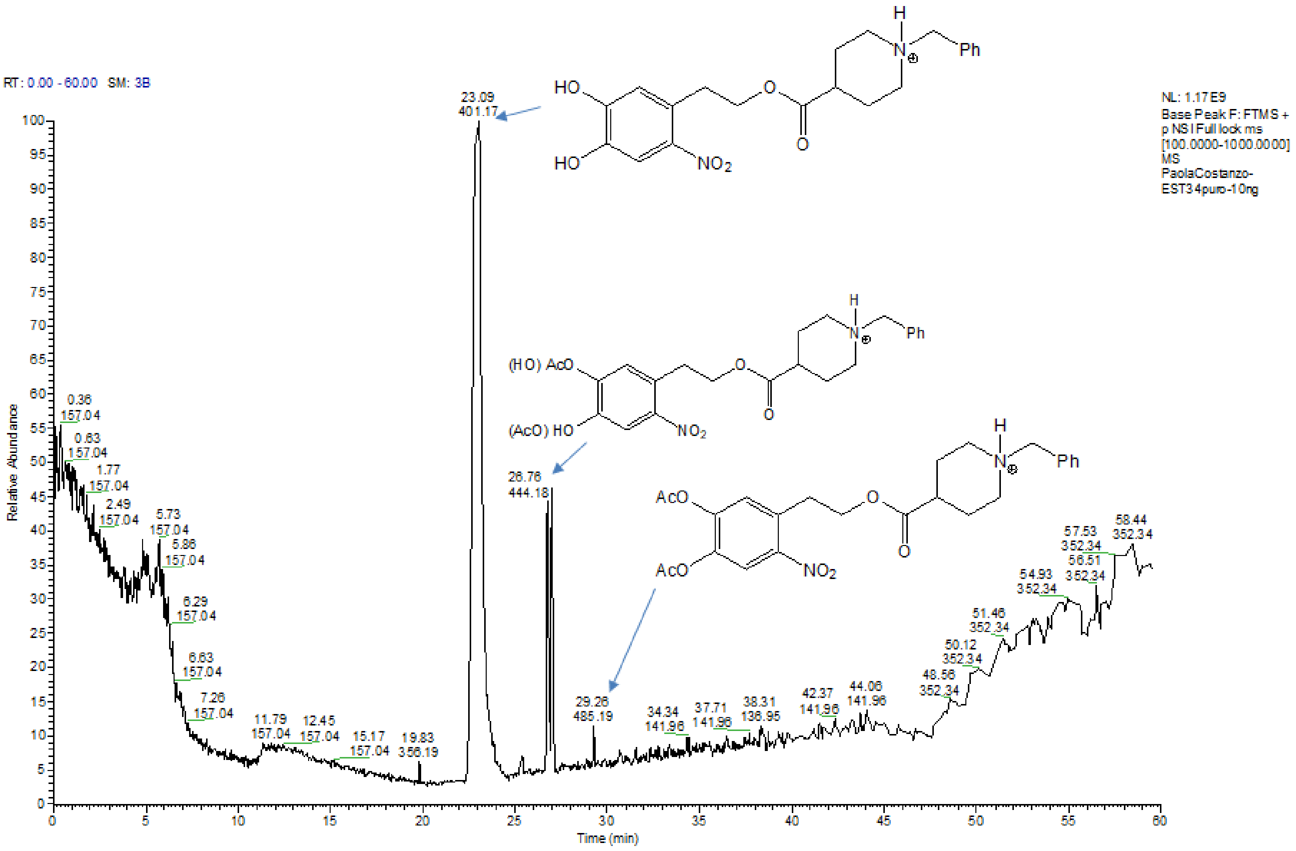

Supplementary Figure 2. Detection of the acetylated and di-acetylated form of **HT2** in the reaction crude. [**HT2-monoacetylated + H]$^+$:** 443.1811 (theoretical [M+H]$^+$ m/z 443.1813); [**HT2-diacetylated + H]$^+$:** 485.1917 (theoretical [M+H]$^+$ m/z 485.1918).

3 Evaluation of pro-oxidant effect for donepezil hybrids

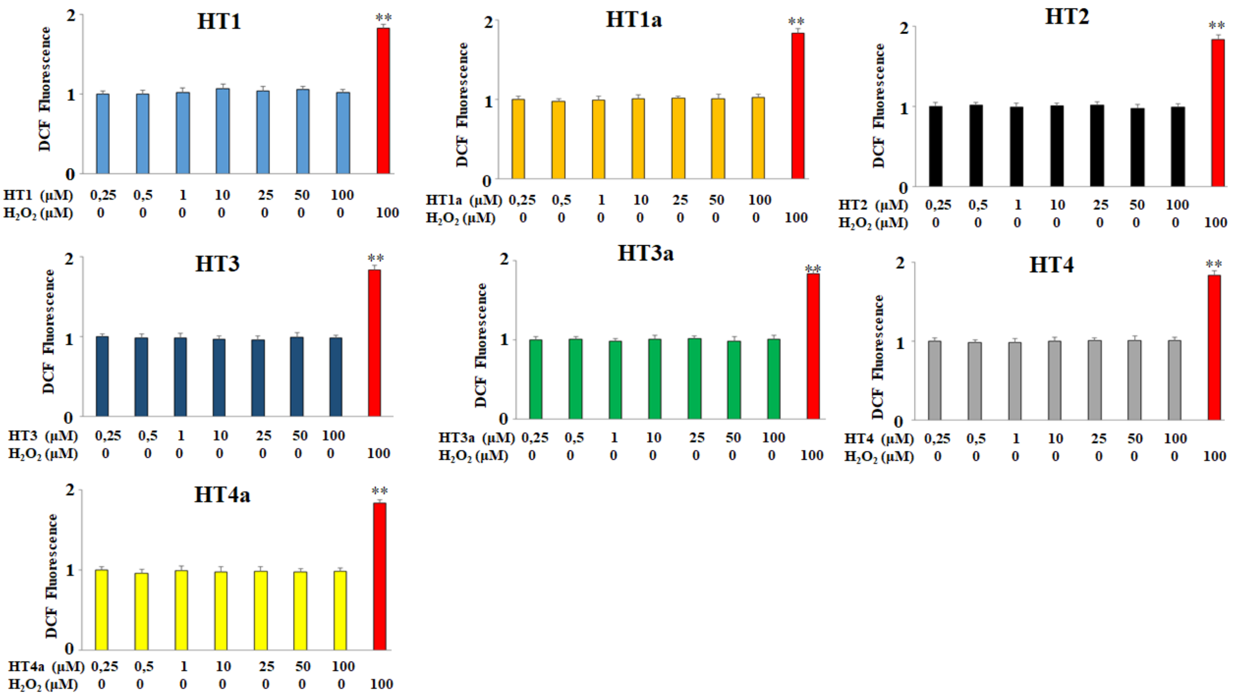

Supplementary Figure 3. Donepezil hybrids have no pro-oxidant effects. Human neuroblastoma (SH-SY5Y) cell line was treated with donepezil hybrids for 24 h at the concentrations shown in the figure. No concentration showed accumulation of ROS. H_2O_2 was used as positive control. Three independent experiments were carried out, with values expressed as the mean ± standard deviation (sd). ** denotes $p < 0.01$ vs. control. Analysis of Variance (ANOVA) was followed by a Tukey–Kramer comparison test.

4 SH-SY5Y Cell viability after treatment with H_2O_2

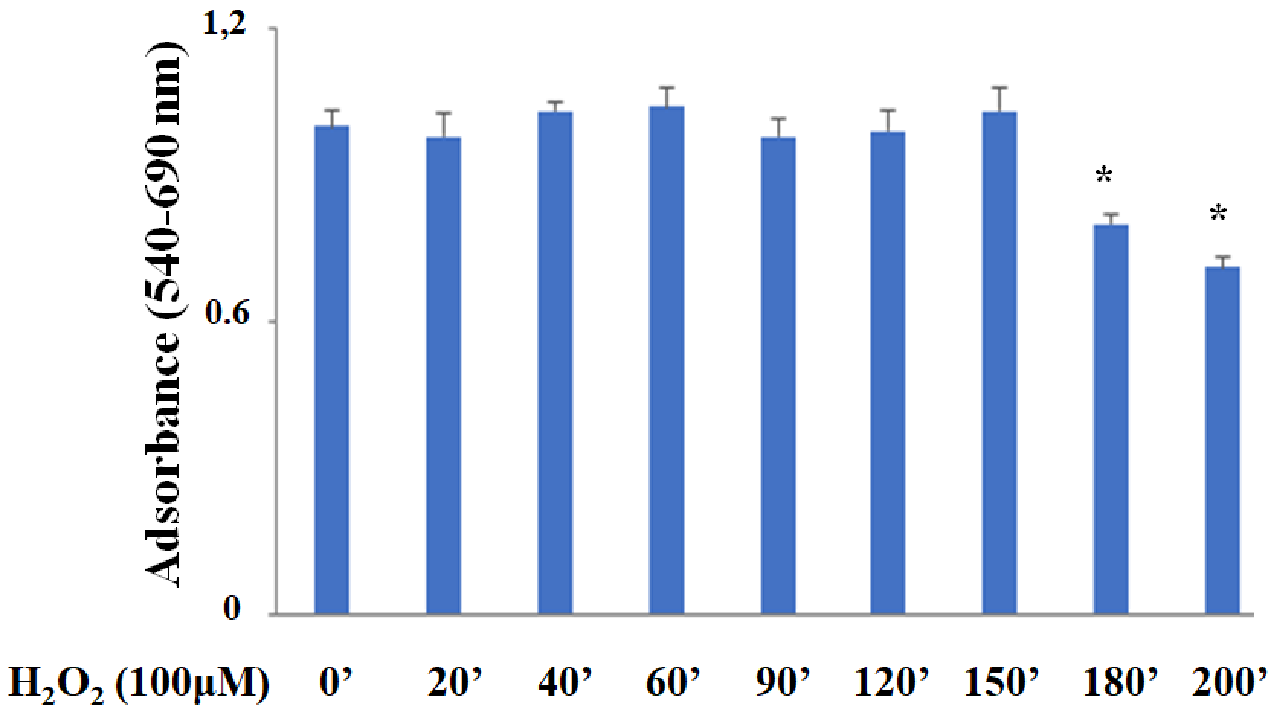

Supplementary Figure 4. Measurement of cell viability following treatment with H_2O_2. Cell viability following treatment with H_2O_2 100µM was determined for several times (0-200'). The values of three independent experiments are expressed as the means ± standard deviations. * denotes $p < 0.05$ vs. control. Analysis of Variance (ANOVA) was followed by the Tukey–Kramer comparison test.

5 CUPRAC test

The CUPRAC test was performed as reported by Ozyürek et al. (Ozyürek et al., 2008) on the new compounds in the unacetylated form.

Supplementary Table 1. Concentration linearity range for all the unprotected hybrids.

Antioxidant	Linear equation and correlation coefficient	ε (L mol−1 cm−1)	TEAC
Trolox	A= 0,0133, c - 0,0414, r = 0.9979	0,0133	1
HT	A= 0,0375, c - 0,0201, r =0,9991	0,0375	2,82
HT1	A= 0,0447, c - 0,0336, r = 0,9943	0,0447	3,36
HT2	A= 0,0369, c+ 0,0178, r = 0,998	0,0369	2,77
HT3	A= 0,0239, c - 0,0192, r = 0,9945	0,0239	1,80
HT4	A= 0,0132, c - 0,0164, r = 0.9948	0,0132	0,99

6 Characterization of compounds

3,4-dihydroxyphenethyl 1-benzylpiperidine-4-carboxylate (HT1): White power, Yield 65%, R_f= (DCM:MeOH:TEA – 84:13:2) 0.6. HRMS: $[M+H]^+$ m/z 356.1852 (theoretical $[M+H]^+$ m/z 356.1856). ^{1}H NMR δ (ppm) (500 MHz, d_6-DMSO): 8.64-8.71 (m br, 2H, OH), 7.21-7.33 (m, 5H), 6.63 (d, *J*=8 Hz, 1H), 6.59 (d, 1H, *J*=2 Hz, 1H), 6.45 (dd, *J*=8 Hz and 2 Hz, 1H), 4.13 (t, *J*=7.0 Hz, 2H), 3.43 (s, 2H), 2.66-2.72 (m, 4H), 2.22-2.36 (m, 1H), 1.96-2.01 (t, *J*=11.2 Hz, 2H), 1.72-1.76 (m, 2H), 1.49-1.57(m, 2H). ^{13}C NMR (500 MHz, d_6-DMSO): δ 174.16, 144.96, 143.60, 128.46, 128.48, 128.02, 126.73, 119.36, 116.11, 115.34, 64.51, 62.13, 52.03, 45.63, 33.70, 27.81.

4,5-dihydroxy-2-nitrophenethyl 1-benzylpiperidine-4-carboxylate (HT2): Orange powde r, Yield=85%, R_f= ($CHCl_3$:AcOEt:TEA-60:38:2) 0.56. HRMS: $[M+H]^+$ m/z 440,2058 (theoretical $[M+H]^+$ m/z 440.2068). ^{1}H NMR δ (ppm) (300MHz, d_6-DMSO): 7.47 (s, 1H), 7.21-7.33 (m, 6H), 4.23 (t, *J*=6.3 Hz, 2H), 3.42 (s, 2H,), 3.08 (t, *J*=6.3 Hz, 2H), 2.69 (br d, *J*=10.7 Hz, 2H), 2.18-2.25 (m, 1H), 1.96 (br t, *J*=9.3 Hz, 2H), 1.70-1.73 (m, 2H), 1.46-1.53 (m, 2H); ^{13}C NMR (500 MHz, d_6-DMSO). 174.16, 152.53, 144.35, 138.71, 138.34, 128.74, 128.12, 126.83, 126.75, 118.54, 111.85, 63.29, 62.22, 52.15, 32.15, 30.40, 29.22, 27.85.

4-hydroxy-3-methoxyphenethyl 1-benzylpiperidine-4-carboxylate (HT3): White power, Yield=45%, R_f= (CH_2Cl_2:MeOH:TEA – 90:8:2) 0.70. HRMS: $[M+H]^+$ m/z 370.2007 (theoretical $[M+H]^+$ m/z 370.2013). ^{1}H NMR δ (ppm) (500 MHz, $CDCl_3$): 7.20-7.32 (m, 5H), 6.83 (d, *J*=7.8 Hz, 1H), 6.68-6.71 (m, 2H), 4.25 (t, *J*=6.9 Hz, 2H), 3.86 (s, 3H), 3.48 (s, 2H), 2.80-2.87 (m, 2H), 2.24-2.31 (m, 1H), 2.00-2.03 (m, 2H), 1.83-1.86 (m, 2H), 1.70-1.79 (m, 2H). ^{13}C NMR (500 MHz, $CDCl_3$): 175.31, 146.68, 144.53, 129.8, 129.34, 128.5, 127.25, 121.84, 114.58, 111.60, 65.21, 63.41, 56.09, 41.36, 35.04, 32.12, 28.42.

4-hydroxyphenethyl 1-benzylpiperidine-4-carboxylate (HT4): White power, Yield=55%; R_f= (DCM:MeOH:TEA – 90:8:2) 0.37. HRMS: $[M+H]^+$ m/z 340.1898 (theoretical $[M+H]^+$ m/z 340.1907). ^{1}H NMR δ (ppm) (500 MHz, $CDCl_3$): 7.23-7.32 (m, 5H), 7.03 (d, *J*=8.4 Hz, 2H), 6.72 (d, *J*=8.4 Hz, 2H), 4.23 (t, *J*=6.9 Hz, 2H), 3.50 (s, 2H), 2.83 (t, *J*=6.9 Hz, 4H), 2.23-2.33 (m, 1H), 2.00-2.08 (m, 2H), 1.82-1.86 (m, 2H), 1.70-1.79 (m, 2H). ^{13}C NMR (500 MHz, $CDCl_3$,): δ 175.31, 154.89, 137.84, 130.22, 129.68, 129.57, 128.46, 127.39, 115.68, 65.31, 63.40, 52.95, 41.23, 34.46, 32.12, 28.16.

4-(2-(1-benzylpiperidine-4-carbonyloxy)ethyl)-1,2-phenylene diacetate (HT1a): Orange oil, Yield=55%, R_f= (CH_2Cl_2:MeOH:TEA-90:8:2) 0.52. HRMS: $[M+H]^+$ m/z 401.1706 (theoretical $[M+H]^+$ m/z 401.1707). ^{1}H NMR δ (ppm) ($CDCl_3$, 300 MHz): 7.22-7.37 (m, 5H), 7.08-7.18 (m, 2H), 7.00-7.08 (m, 1H), 4.29 (t, *J*=9.9 Hz, 2H), 3.50 (s, 2H), 2.92 (t, *J*=9.9 Hz, 2H), 2.84 (d, *J*=11 Hz, 2H), 2.22-2.38 (m, 7H), 1.92-2.10 (m, 2H), 1.90-1.65 (m, 4H); ^{13}C NMR ($CDCl_3$, 300 MHz). δ: 179.1, 175.2, 168.4, 146.3, 142.5, 137.8, 135.3, 129.9, 128.7, 127.5, 124.3, 65.4, 63.3, 52.4, 41.9, 34.6, 28.1, 20.7.

4-acetoxy-3-methoxyphenethyl 1-benzylpiperidine-4-carboxylate (HT3a): Orange oil, Yield=98%, R_f= (CH_2Cl_2:MeOH:TEA – 90:8:2) 0.77; HRMS: $[M+H]^+$ m/z 412.2111 (theoretical $[M+H]^+$ m/z 412.2118). ^{1}H NMR δ (ppm) (500 MHz, $CDCl_3$): 7.15-7.24 (m, 5H), 6.87 (d, *J*=8.0 Hz,

1H), 6.69-6.75 (td, *J*=8.0 and *J*=1.8 Hz, 2H), 4.22 (t, *J*=7.2 Hz, 2H), 3.74 (s, 3H), 3.41 (s, 2H), 2.84 (t, *J*=7.0 Hz, 2H), 2.75 (br d, *J*=11.0 Hz, 2H), 2.24 (s, 3H), 2.16-2.22 (m, 1H), 1.94 (t br, =11.0 Hz, 2H), 1. 77-1.79 (m, 2H), 1.63-1.73 (m, 2H). ^{13}C NMR ($CDCl_3$, 500 MHz): 175.32, 169.34, 151.13, 138.6, 136.99, 129.28, 128.4, 127.19, 122.88, 121.27, 113.27, 64.76, 63.44, 56.07, 53.07, 41.42, 35.26, 28.49, 20.88.

4-acetoxyphenethyl 1-benzylpiperidine-4-carboxylate (HT4a): Incolor oil; Yield=85%; R_f= ($CHCl_3$:AcOEt:TEA-60:38:2) 0.28. HRMS: $[M+H]^+$ m/z 382.2005 (theoretical $[M+H]^+$ m/z 382.2013); ^{1}H NMR δ (ppm) (500 MHz, $CDCl_3$): 7.15-7.24 (m, 5H), 7.14 (d, *J*=8.4 Hz, 2H), 6.94 (d, *J*=8.5 Hz, 2H), 4.21 (t, *J*=6.8 Hz, 2H), 3.41 (s, 2H), 2.85 (t, *J*=6.9 Hz, 2H), 2.73-2.77 (br d, *J*=11.5 Hz, 2H), 2.16-2.23 (m, 4H), 1.91-1.96 (br t, *J*=10.8 Hz, 2H), 1.74-1.81 (m, 2H), 1.61-1.70 (m, 2H); ^{13}C NMR (500 MHz, $CDCl_3$,): δ 175.31, 169.77, 149.54, 138.58, 135.66, 130.08, 129.32, 128.40, 127.19, 121.75, 64.78, 63.45, 53.08, 41.37, 34.72, 28.1, 21.35.

7 HRMS

HT1

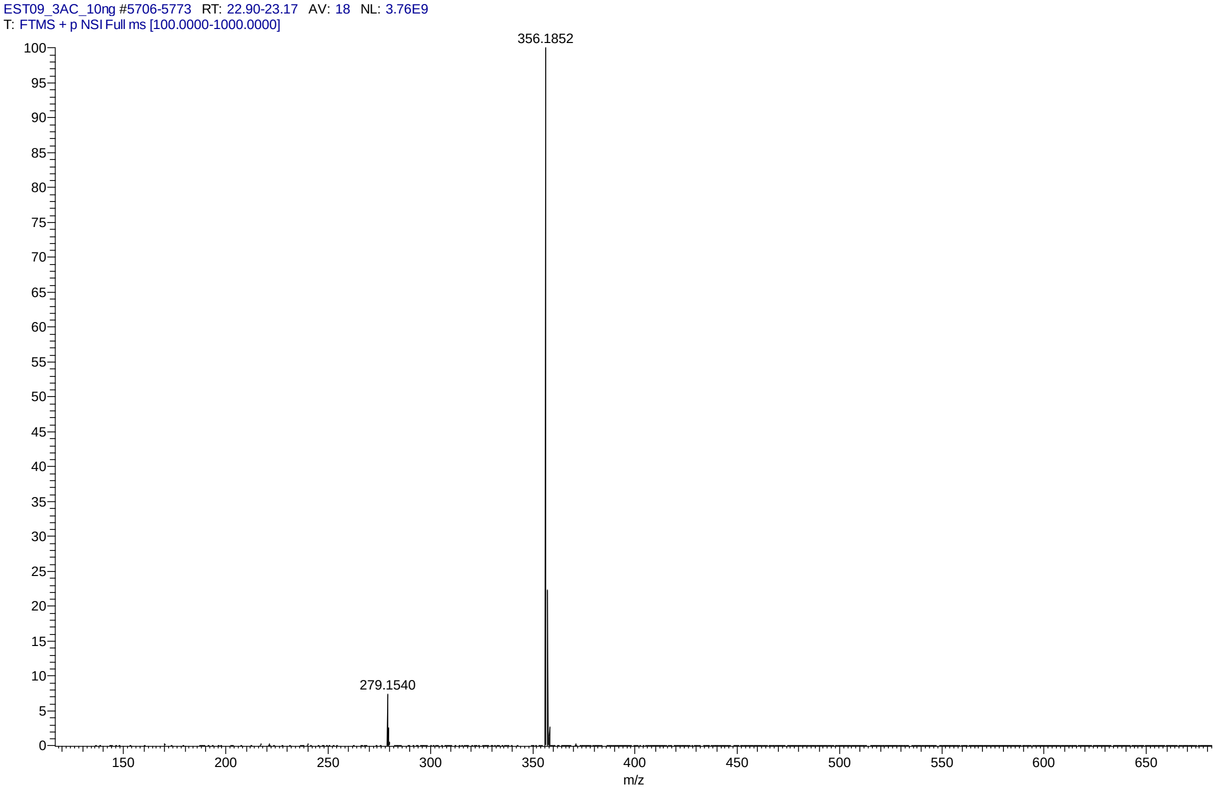

HT2

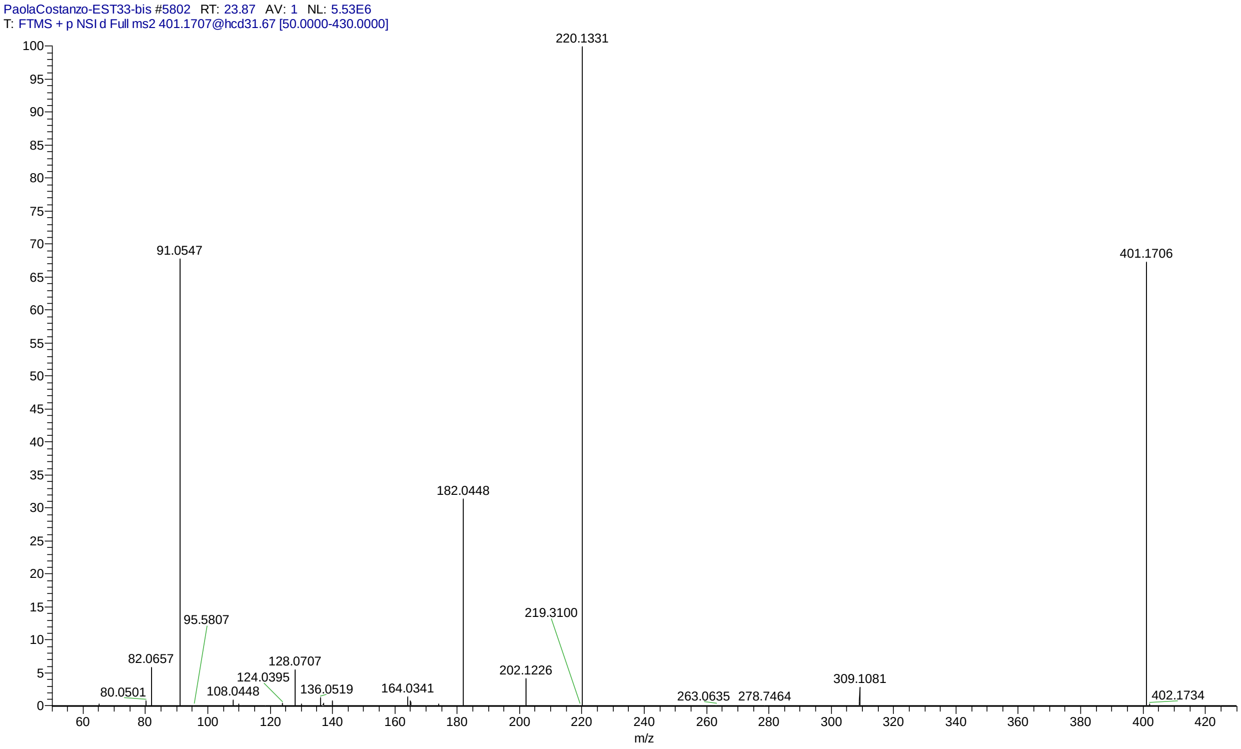

HT3

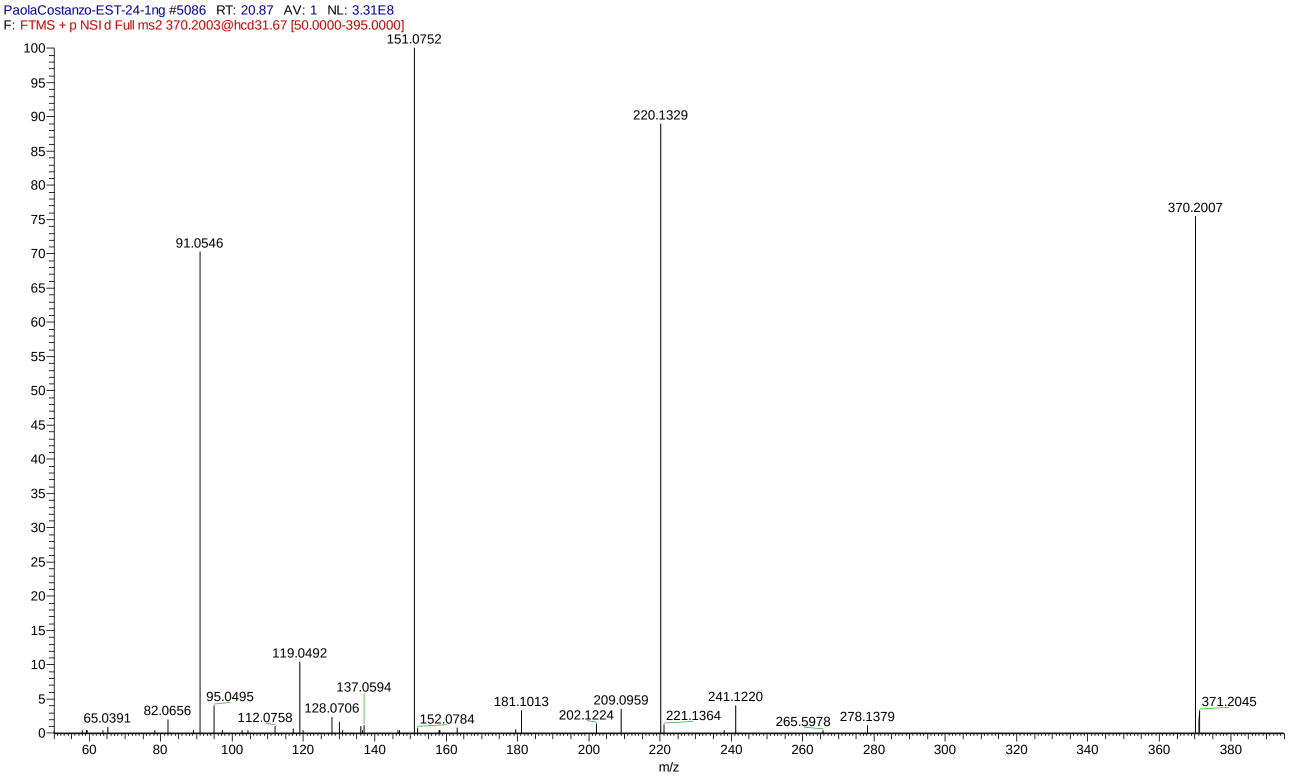
PaolaCostanzo-EST-24-1ng #5086 RT: 20.87 AV: 1 NL: 3.31E8
F: FTMS + p NSI d Full ms2 370.2003@hcd31.67 [50.0000-395.0000]
151.0752
220.1329
370.2007
91.0546
119.0492
137.0594
95.0495
181.1013
209.0959
241.1220
371.2045
65.0391
82.0656
112.0758
128.0706
152.0784
202.1224
221.1364
265.5978
278.1379
m/z

HT4

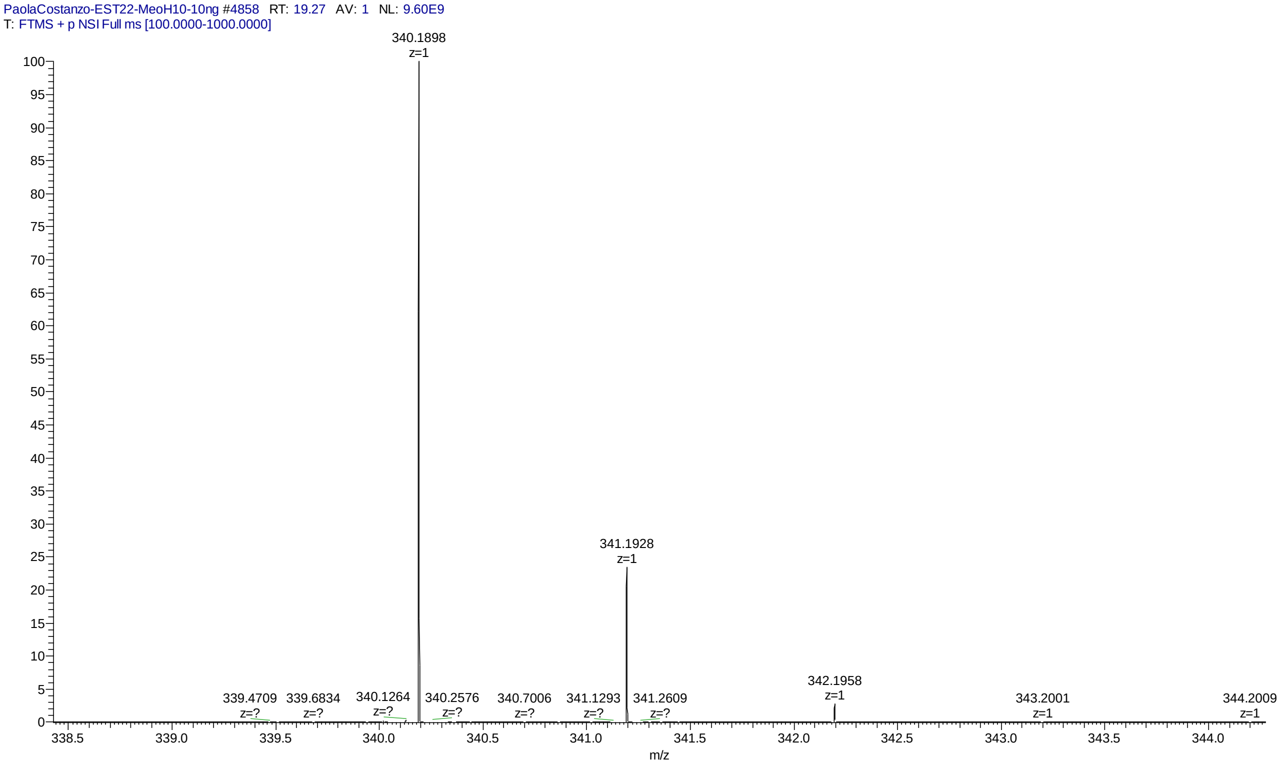
PaolaCostanzo-EST22-MeoH10-10ng #4858 RT: 19.27 AV: 1 NL: 9.60E9
T: FTMS + p NSI Full ms [100.0000-1000.0000]
340.1898
z=1
341.1928
z=1
342.1958
z=1
339.4709
z=?
339.6834
z=?
340.1264
z=?
340.2576
z=?
340.7006
z=?
341.1293
z=?
341.2609
z=?
343.2001
z=1
344.2009
z=1
m/z

HT1a

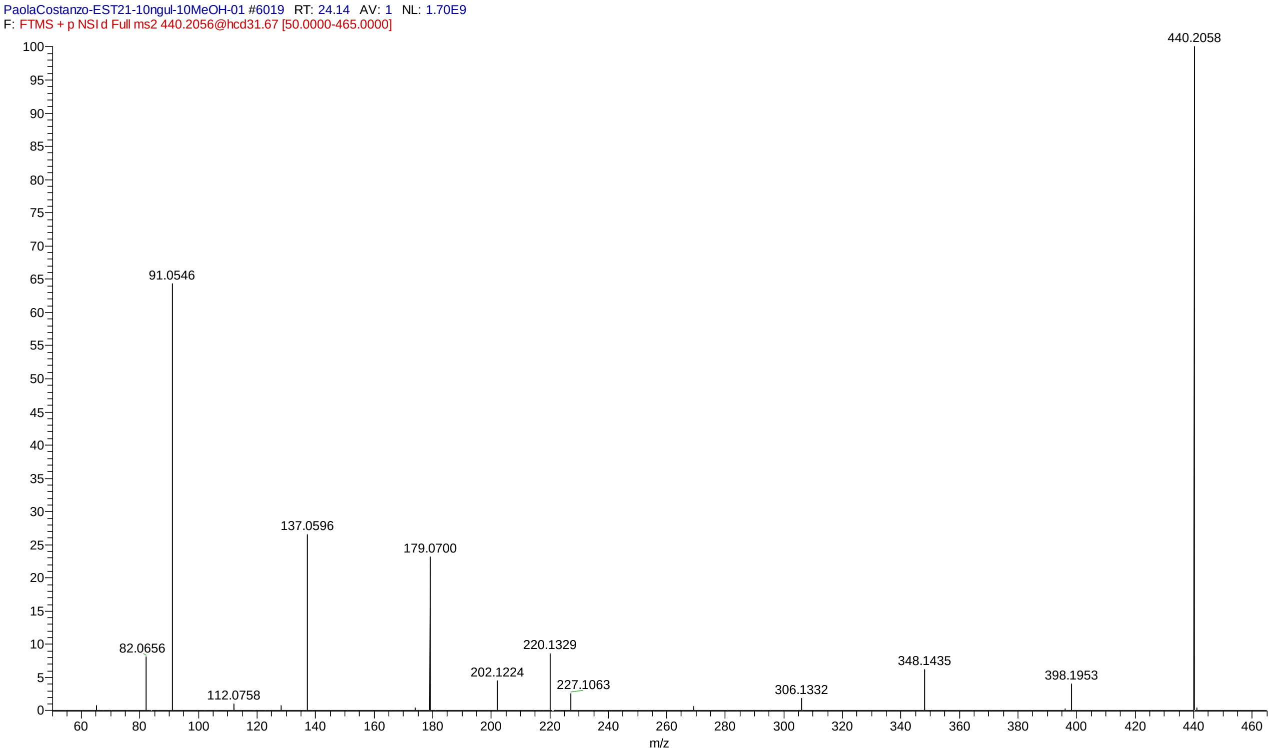

PaolaCostanzo-EST21-10ngul-10MeOH-01 #6019 RT: 24.14 AV: 1 NL: 1.70E9
F: FTMS + p NSI d Full ms2 440.2056@hcd31.67 [50.0000-465.0000]
440.2058
91.0546
137.0596
179.0700
220.1329
82.0656
202.1224
227.1063
112.0758
306.1332
348.1435
398.1953
m/z

HT3a

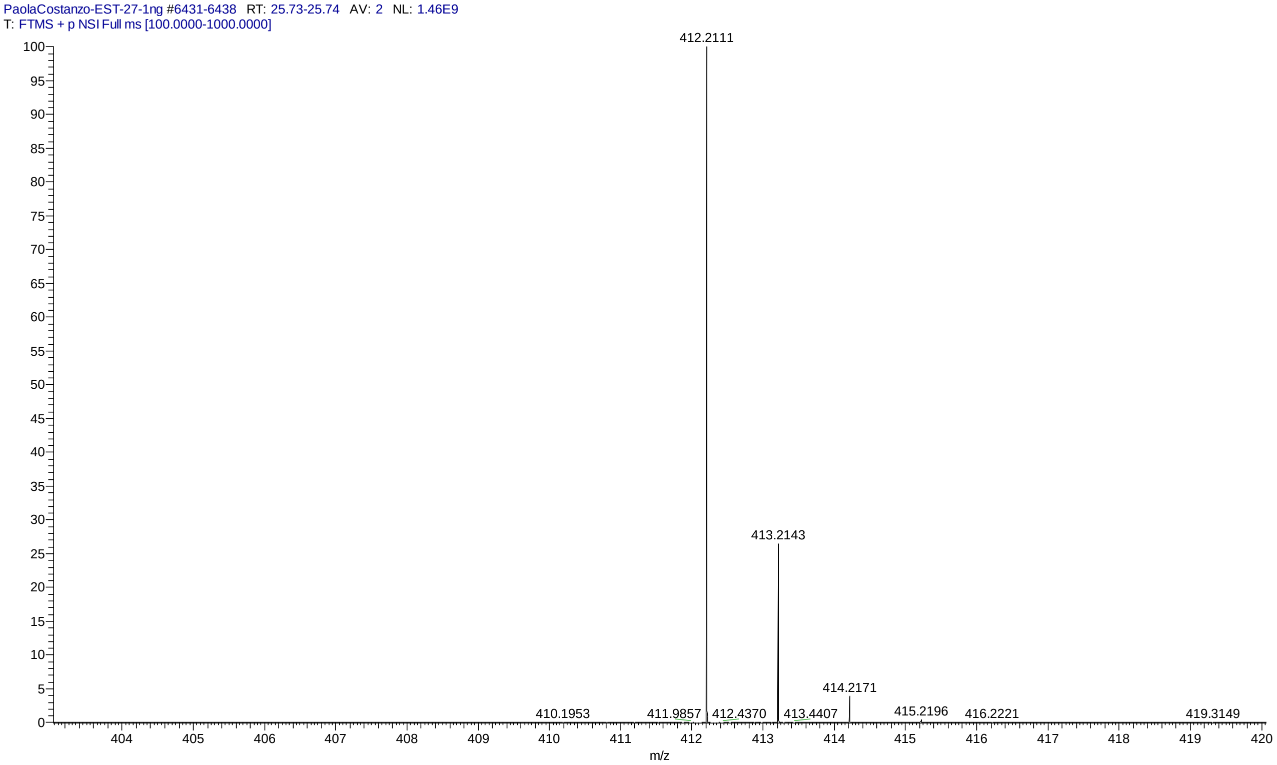

PaolaCostanzo-EST-27-1ng #6431-6438 RT: 25.73-25.74 AV: 2 NL: 1.46E9
T: FTMS + p NSI Full ms [100.0000-1000.0000]
412.2111
413.2143
414.2171
410.1953
411.9857
412.4370
413.4407
415.2196
416.2221
419.3149
m/z

8 ^{1}H-NMR

HT1

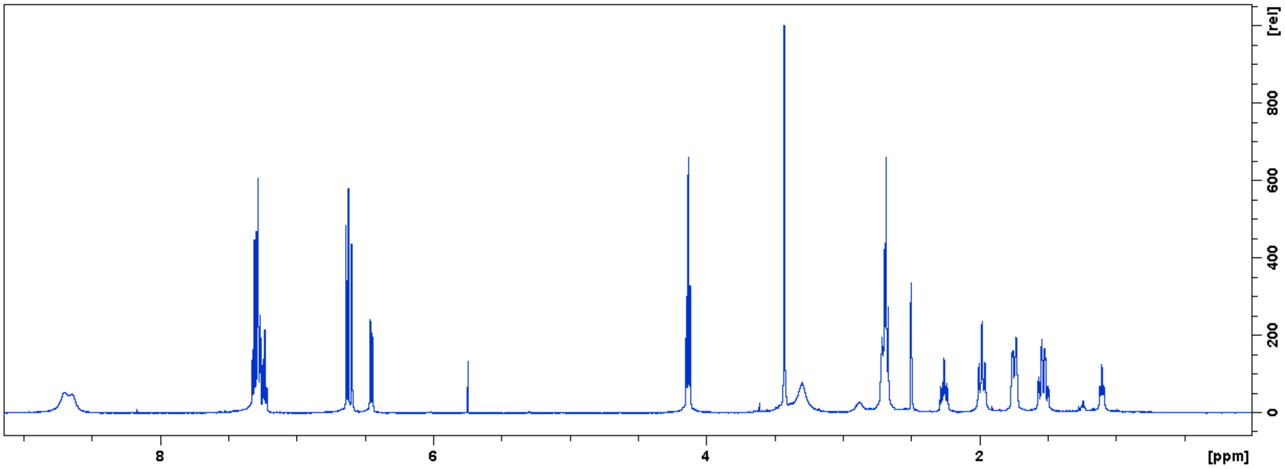

HT2

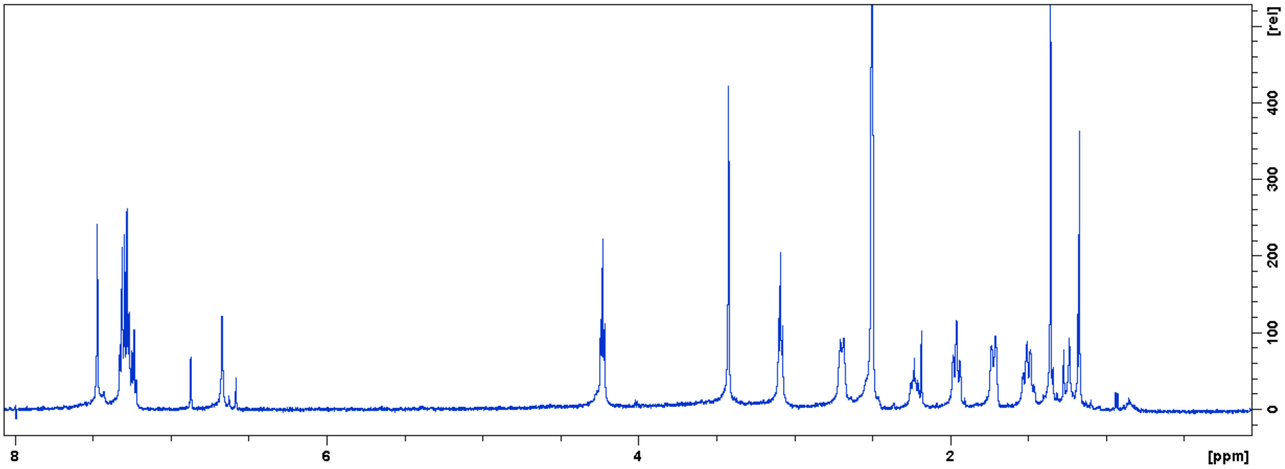

HT3

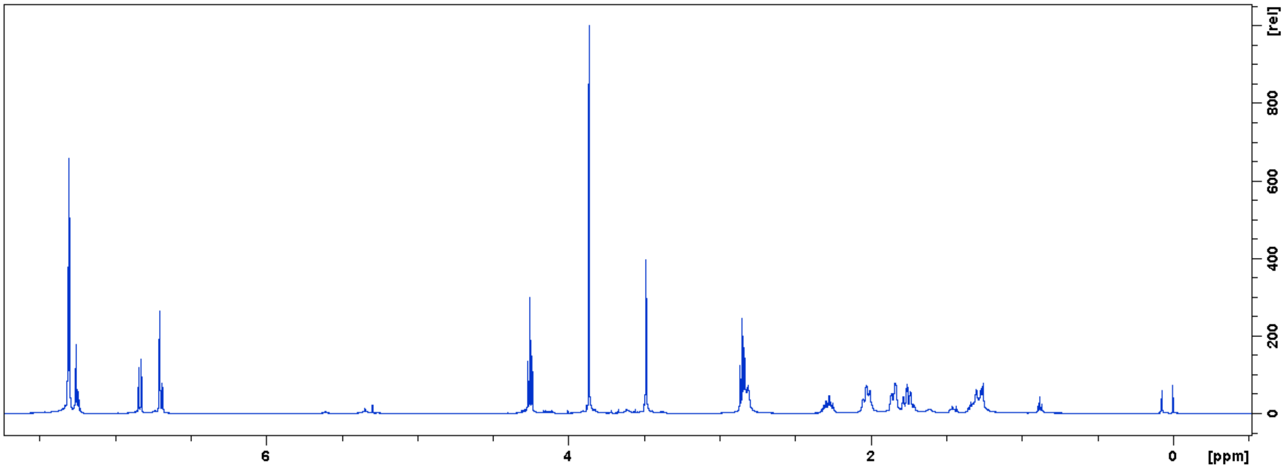

HT4

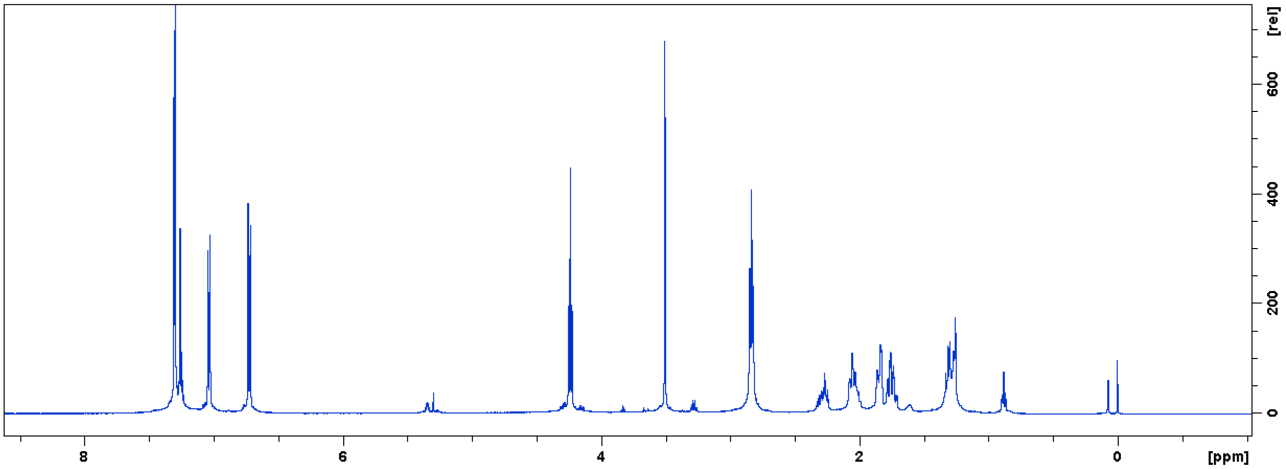
[rel]
0
200
400
600
8
6
4
2
0
[ppm]

HT1a

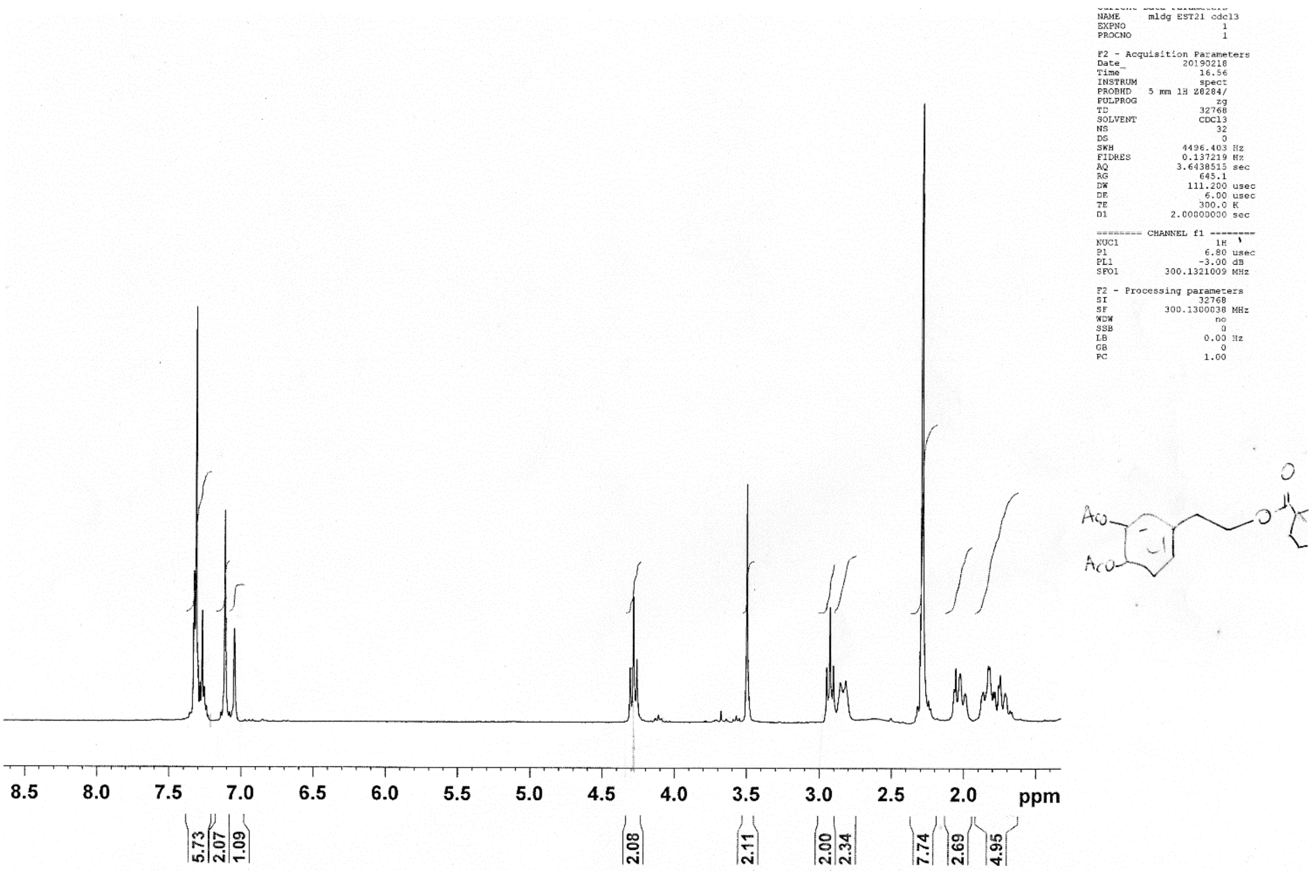
NAME mldg EST21 cdcl3
EXPNO 1
PROCNO 1
F2 - Acquisition Parameters
Date_ 20190218
Time 16.56
INSTRUM spect
PROBHD 5 mm 1H Z8284/
PULPROG zg
TD 32768
SOLVENT CDCl3
NS 32
DS 0
SWH 4496.403 Hz
FIDRES 0.137219 Hz
AQ 3.6438515 sec
RG 645.1
DW 111.200 usec
DE 6.00 usec
TE 300.0 K
D1 2.00000000 sec
======== CHANNEL f1 ========
NUC1 1H
P1 6.80 usec
PL1 -3.00 dB
SFO1 300.1321009 MHz
F2 - Processing parameters
SI 32768
SF 300.1300038 MHz
WDW no
SSB 0
LB 0.00 Hz
GB 0
PC 1.00
8.5
8.0
7.5
7.0
6.5
6.0
5.5
5.0
4.5
4.0
3.5
3.0
2.5
2.0
ppm
5.73
2.07
1.09
2.08
2.11
2.00
2.34
7.74
2.69
4.95

HT3a

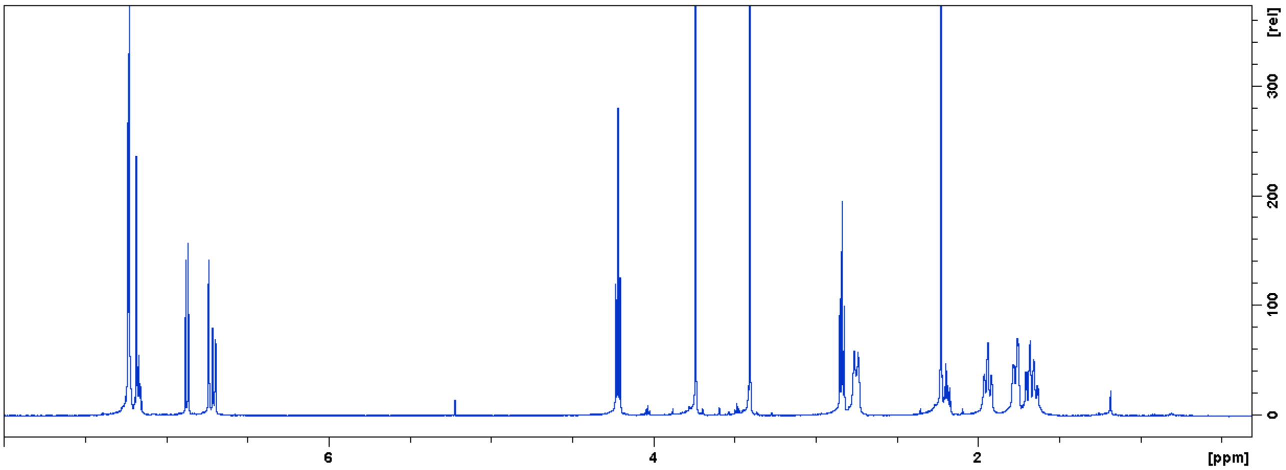

HT4a

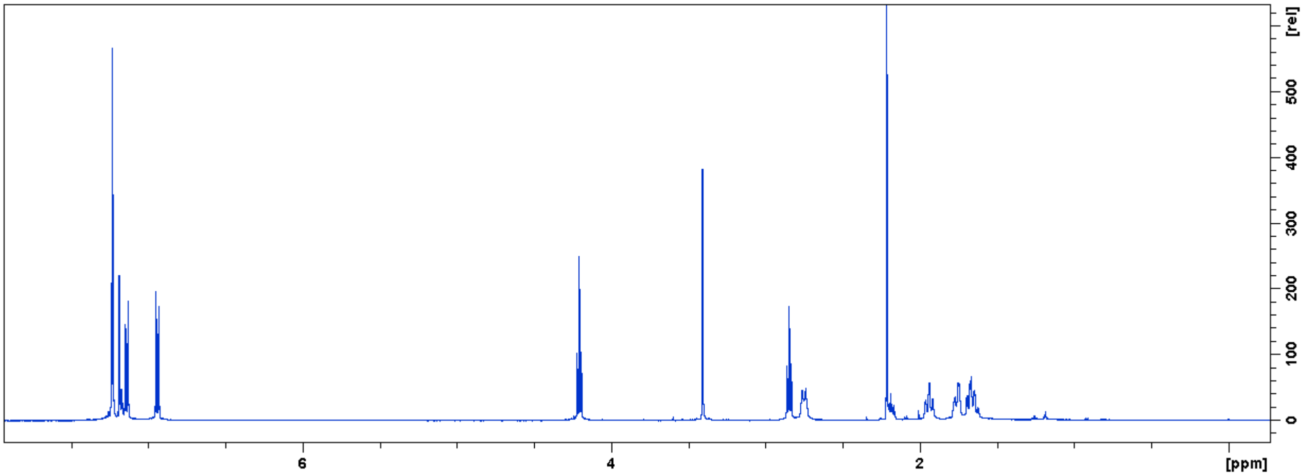

9 ^{13}C-NMR

HT1

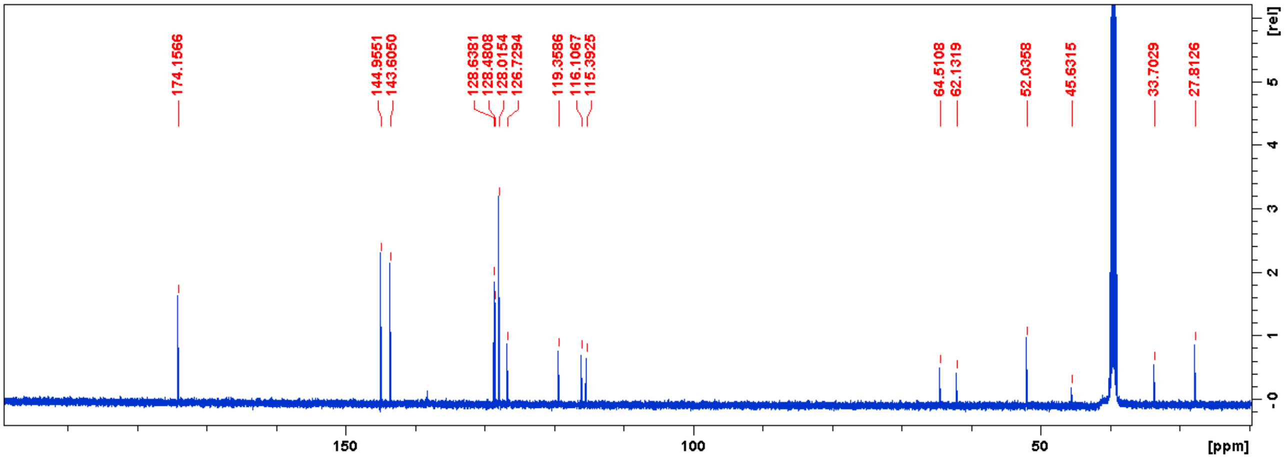

HT2

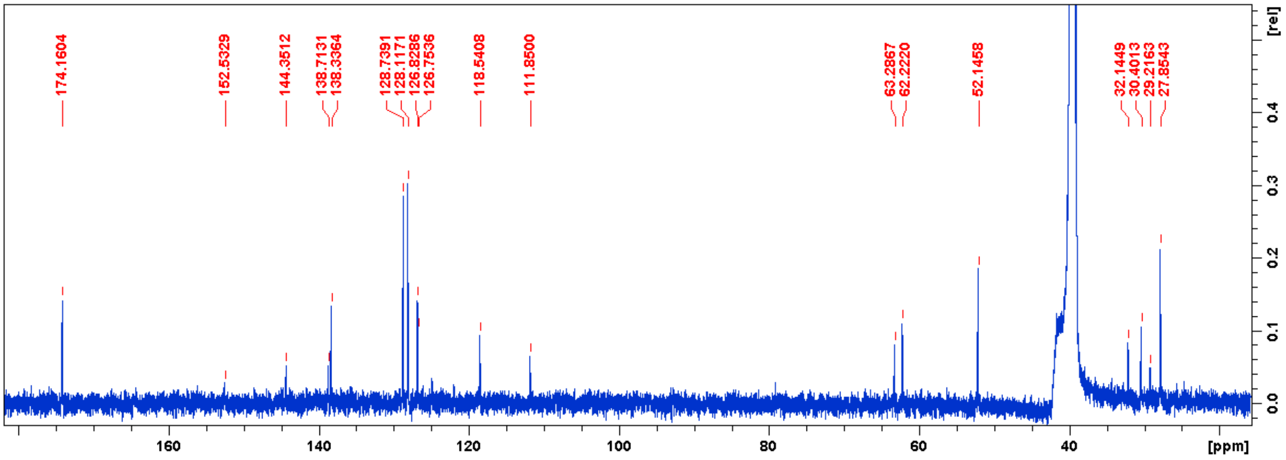

HT3

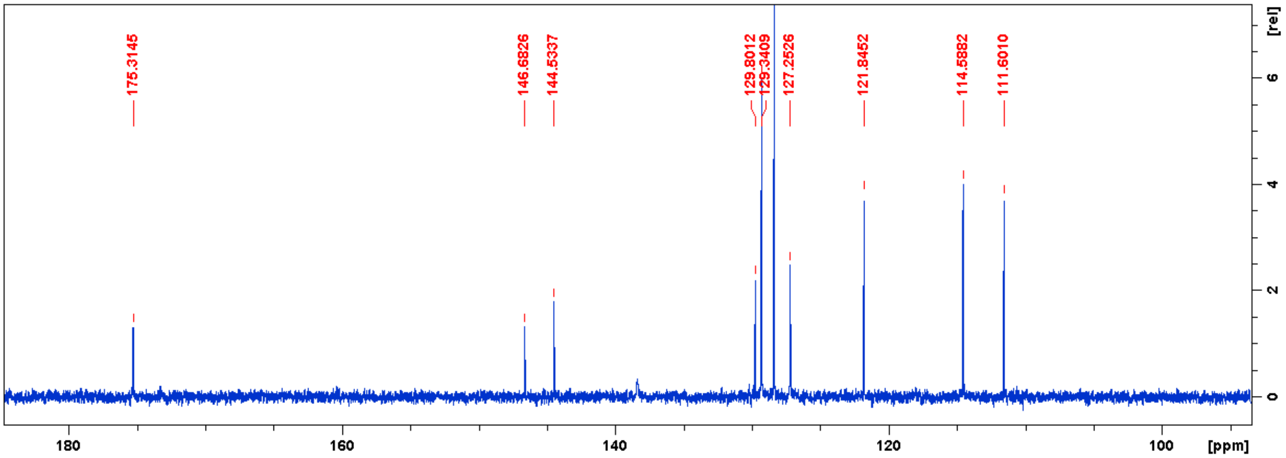

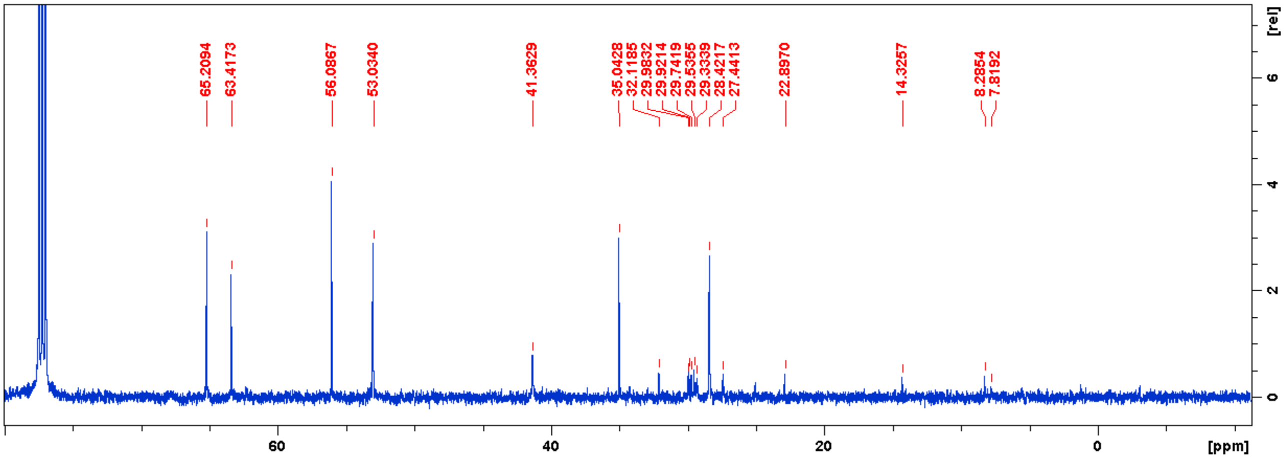
65.2094
63.4173
56.0867
53.0340
41.3629
35.0428
32.1185
29.9832
29.9214
29.7419
29.5355
29.3339
28.4217
27.4413
22.8970
14.3257
8.2854
7.8192
[rel]
60
40
20
0
[ppm]

HT4

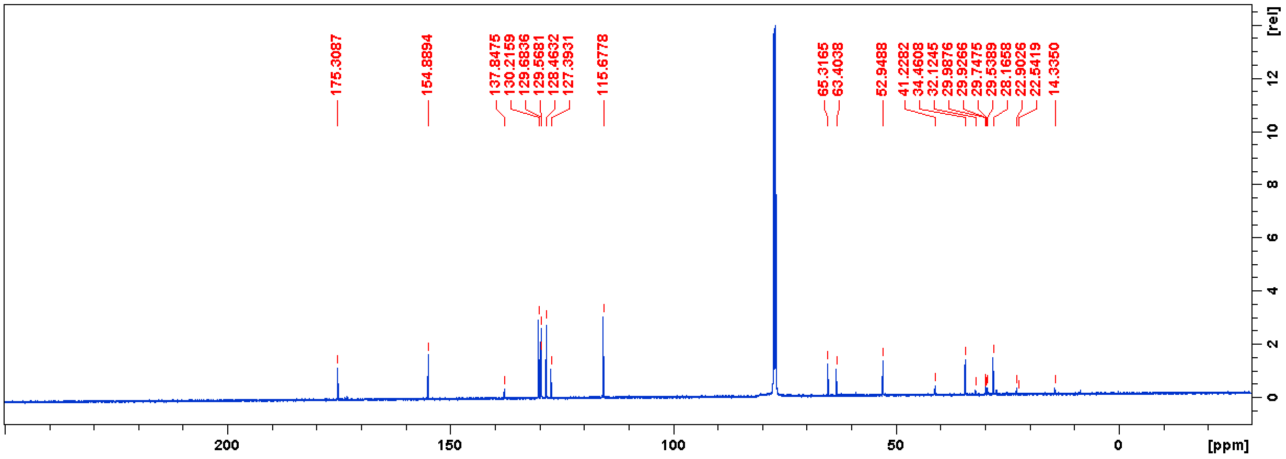
175.3087
154.8894
137.8475
130.2159
129.6836
129.5681
128.4632
127.3931
115.6778
65.3165
63.4038
52.9488
41.2282
34.4608
32.1245
29.9876
29.9266
29.7475
29.5389
28.1658
22.9026
22.5419
14.3350
[rel]
200
150
100
50
0
[ppm]

HT1a

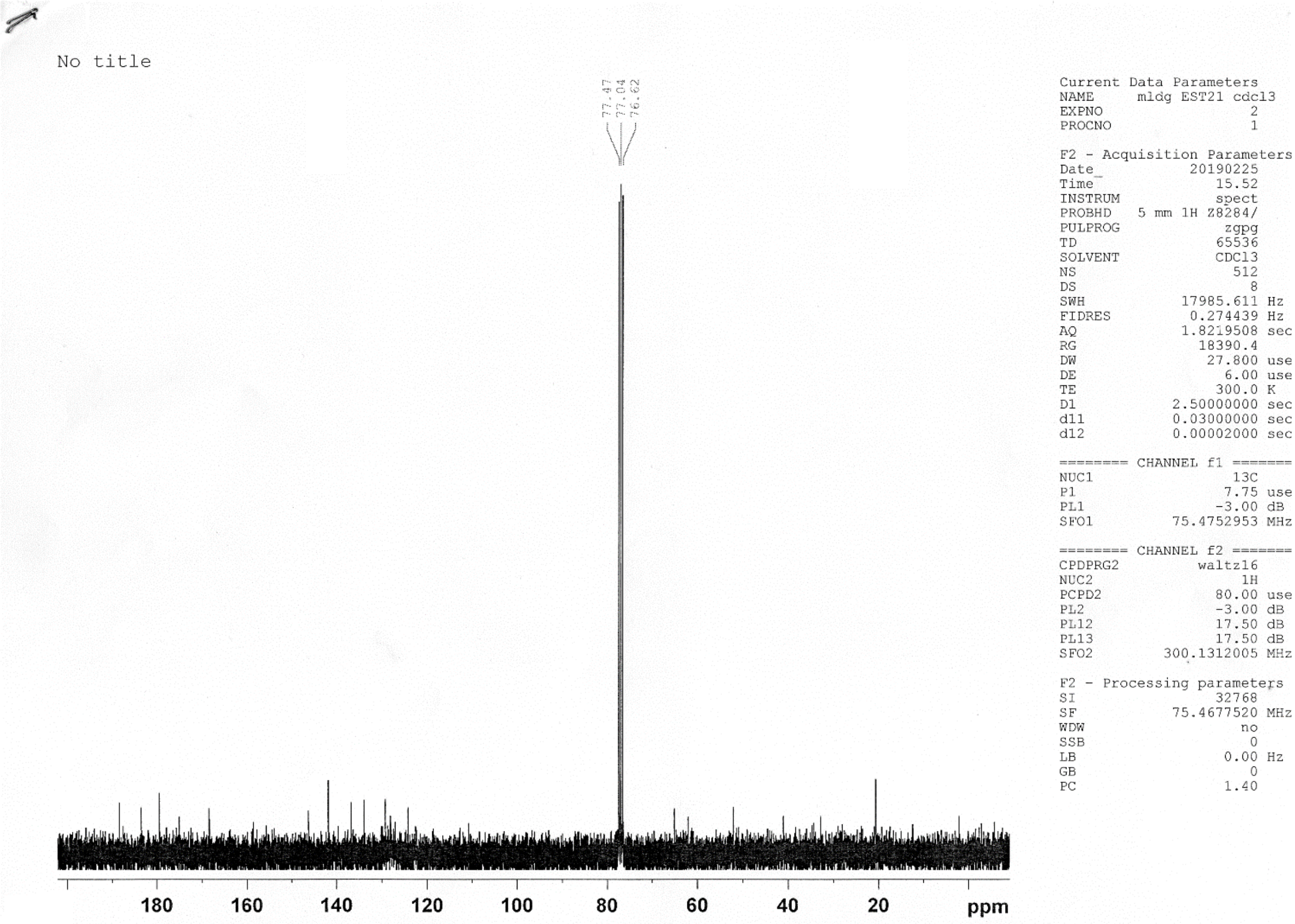
No title
77.47
77.04
76.62
Current Data Parameters
NAME mldg EST21 cdcl3
EXPNO 2
PROCNO 1
F2 - Acquisition Parameters
Date_ 20190225
Time 15.52
INSTRUM spect
PROBHD 5 mm 1H Z8284/
PULPROG zgpg
TD 65536
SOLVENT CDCl3
NS 512
DS 8
SWH 17985.611 Hz
FIDRES 0.274439 Hz
AQ 1.8219508 sec
RG 18390.4
DW 27.800 use
DE 6.00 use
TE 300.0 K
D1 2.50000000 sec
d11 0.03000000 sec
d12 0.00002000 sec
======== CHANNEL f1 =======
NUC1 13C
P1 7.75 use
PL1 -3.00 dB
SFO1 75.4752953 MHz
======== CHANNEL f2 =======
CPDPRG2 waltz16
NUC2 1H
PCPD2 80.00 use
PL2 -3.00 dB
PL12 17.50 dB
PL13 17.50 dB
SFO2 300.1312005 MHz
F2 - Processing parameters
SI 32768
SF 75.4677520 MHz
WDW no
SSB 0
LB 0.00 Hz
GB 0
PC 1.40
180
160
140
120
100
80
60
40
20
ppm

HT3a

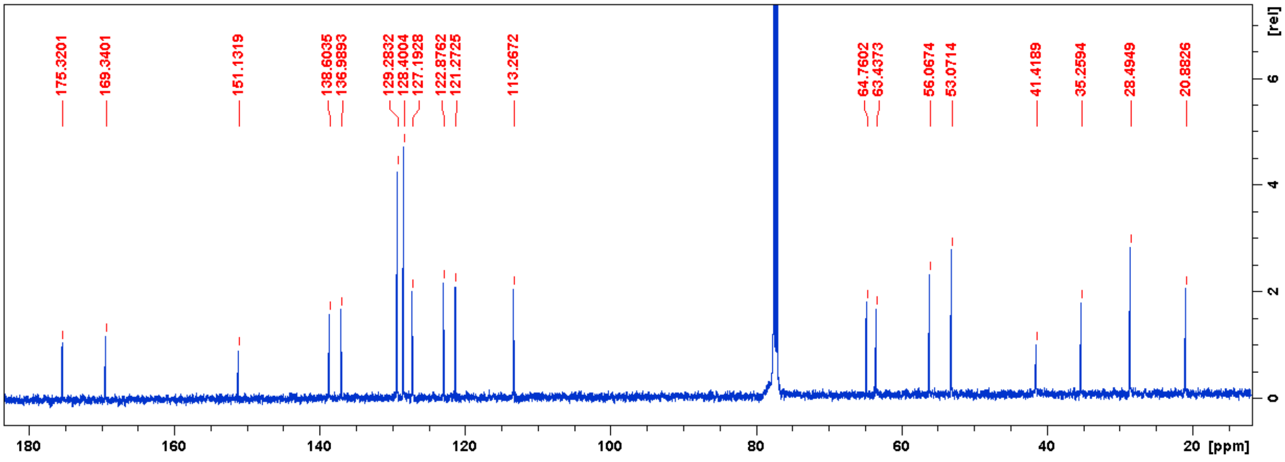
175.3201
169.3401
151.1319
138.6035
136.9893
129.2832
128.4004
127.1928
122.8762
121.2725
113.2672
64.7602
63.4373
56.0674
53.0714
41.4189
35.2594
28.4949
20.8826
[rel]
6
4
2
0
180
160
140
120
100
80
60
40
20 [ppm]

HT4a

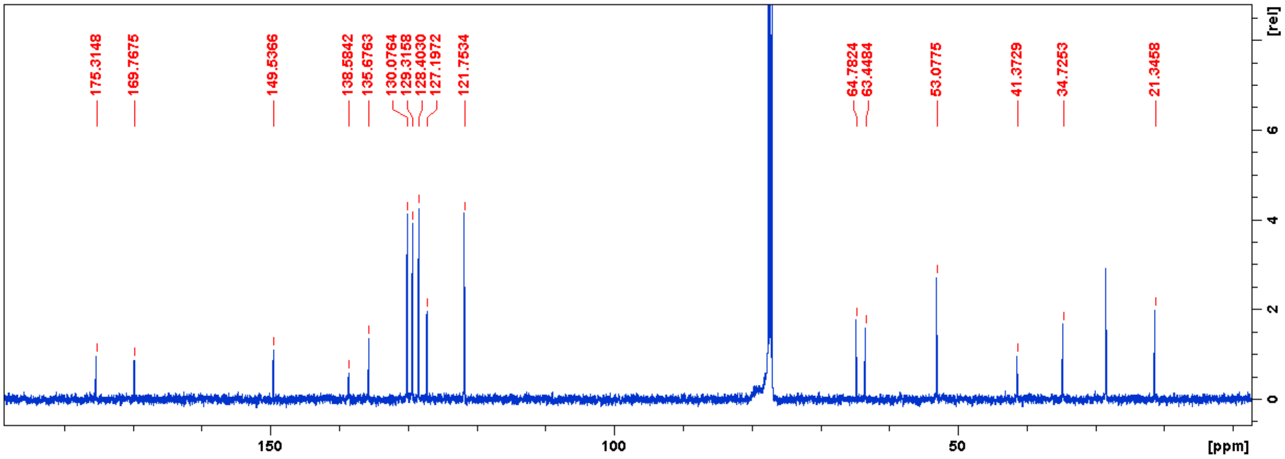

10 References

Ou, B., Hampsch-Woodill, M., and Prior, R. L. (2001). Development and validation of an improved oxygen radical absorbance capacity assay using fluorescein as the fluorescent probe, *J. Agric. Food Chem*. 49, 4619–4626. doi: 10.1021/jf010586o.

Ozyürek, M., Bektaşoğlu, B., Güçlü, K., Güngör, N., and Apak, R. (2008). Simultaneous total antioxidant capacity assay of lipophilic and hydrophilic antioxidants in the same acetone-water solution containing 2% methyl-beta-cyclodextrin using the cupric reducing antioxidant capacity (CUPRAC) method. *Anal. Chim. Acta*. 630(1), 28-39. doi: 10.1016/j.aca.2008.09.057.
